# Hybrid evolution repeats itself across environmental contexts in Texas sunflowers (*Helianthus*)

**DOI:** 10.1111/evo.14536

**Published:** 2022-06-17

**Authors:** Nora Mitchell, Hoang Luu, Gregory L. Owens, Loren H. Rieseberg, Kenneth D. Whitney

**Affiliations:** ^1^ Department of Biology University of New Mexico Albuquerque New Mexico USA; ^2^ Department of Biology University of Wisconsin – Eau Claire Eau Claire Wisconsin USA; ^3^ Department of Environmental and Plant Biology Ohio University Athens Ohio USA; ^4^ Department of Biology University of Victoria Victoria British Columbia Canada; ^5^ Department of Botany and Biodiversity Research Centre University of British Columbia British Columbia Canada

**Keywords:** Convergence, parallel evolution, phenotypic evolution, traits

## Abstract

To what extent is evolution repeatable? Little is known about whether the evolution of hybrids is more (or less) repeatable than that of nonhybrids. We used field experimental evolution in annual sunflowers (*Helianthus*) in Texas to ask the extent to which hybrid evolution is repeatable across environments compared to nonhybrid controls. We created hybrids between *Helianthus annuus* (L.) and *H. debilis* (Nutt.) and grew plots of both hybrids and nonhybrid controls through eight generations at three sites in Texas. We collected seeds from each generation and grew each generation × treatment × home site combination at two final common gardens. We estimated the strength and direction of evolution in terms of fitness and 24 traits, tested for repeated versus nonrepeated evolution, and assessed overall phenotypic evolution across lineages and in relation to a locally adapted phenotype. Hybrids consistently evolved higher fitness over time, while controls did not, although trait evolution varied in strength across home sites. Repeated evolution was more evident in hybrids versus nonhybrid controls, and hybrid evolution was often in the direction of the locally adapted phenotype. Our findings have implications for both the nature of repeatability in evolution and the contribution of hybridization to evolution across environmental contexts.

To what extent is evolution repeatable? At the grand scale, “replaying life's tape” (*sensu* Gould 1990) is not feasible, but investigations into the repeatability of evolution using studies of convergence, experimental evolution, and evolutionary genetics enable us to begin to understand when and how evolution may be repeatable (Lobkovsky and Koonin 2012; Orgogozo 2015; Lässig et al. 2017).

At the macroevolutionary scale, examples of convergent evolution have been used to support the idea that evolution is repeatable to some degree (Schluter et al. 2004; Donoghue 2005; Arendt and Reznick 2008; Losos 2011; Ostevik et al. 2012). It is important to investigate the scale of repeatability (evolution occurring in similar ways across space or time), whether examining the broad phenotype or individual genetic changes. Furthermore, repeatable evolution may be the result of three genetic causes: (1) evolution by novel mutations that occurred independently; (2) evolution via similar changes in the frequency of alleles found in ancestral populations (i.e., evolution from standing variation); or (3) evolution via alleles introduced via hybridization or introgression from a separate population (Stern 2013). Here, we investigate the repeatability of phenotypic evolution in the context of increased genetic variation produced by hybridization.

At the microevolutionary scale, both observational and experimental studies have found evidence for phenotypic or genetic convergence, which are often condition‐dependent (reviewed in Lobkovsky and Koonin 2012; Stern 2013; Orgogozo 2015; Lässig et al. 2017). For example, multiple invasions of freshwater environments by marine stickleback fish resulted in the evolution of similar morphologies (Schluter et al. 2004) as well as similar genetic changes across historic and newly formed populations (Kingman et al. 2021). In plants, for instance, adaptation to serpentine soils has driven similar changes in physiological tolerance to heavy metals as well as additional changes in phenology and floral morphology (reviewed in Brady et al. 2005; Sianta and Kay 2021), while adaptation to coastal habitats has produced the evolution of ecotypes with similar growth forms (James et al. 2021), and adaptation to urban environments has selected against cyanogenesis (likely associated with an increased need for freezing tolerance in urban centers) (Santangelo et al. 2022; Thompson et al. 2016). Some notable examples of experimental evolution have found evidence for repeated evolution, including the >30‐year study of *Escherichia coli*, which demonstrated both convergence and divergence at different scales in response to distinct resources (e.g., Saxer et al. 2010; Lenski 2017), as well as the response of replicate populations of *Drosophila melanogaster* selected for accelerated development in a laboratory setting (Burke et al. 2010), and responses of *Brassica rapa* replicates to experimental drought conditions (Johnson et al. In Press).

Hybridization is another key field in evolutionary biology, with a major question being the extent to which hybridization and/or introgression between taxa act as evolutionary fuel in promoting speciation, evolution, and diversification (Anderson and Stebbins 1954; Barrett and Schluter 2008; Stelkens et al. 2014; Marques et al. 2019; Taylor and Larson 2019) versus the circumstances under which these processes act as homogenizers, reducing diversity or leading to extinction (Rhymer and Simberloff 1996; Wolf et al. 2001; Todesco et al. 2016). At the macroevolutionary scale, hybridization has been linked to evolutionary radiation (Anderson and Stebbins 1954; Stebbins 1959; Barton 2001; Seehausen 2004; Yakimowski and Rieseberg 2014; Berner and Salzburger 2015; Stankowski and Streisfeld 2015; Grant and Grant 2019; Marques et al. 2019; Meier et al. 2019) and potentially diversification (Mitchell and Whitney 2021). Hybridization can also lead to adaptation (Lewontin and Birch 1966; Campbell and Snow 2007; Hovick et al. 2012; Stankowski and Streisfeld 2015; Mitchell et al. 2019) and speciation (Rieseberg 2003; Rieseberg et al. 2007; Soltis and Soltis 2009; Abbott et al. 2013). As a case study, the sunflower genus (*Helianthus*) contains examples of homoploid hybrid speciation, where ancient hybridization events appear to have resulted in multiple unique hybrid species (reviewed in Rieseberg et al. 2007), while experimental evolution work in another part of the genus found that hybridization increased the speed of evolution (when compared to nonhybrids) (Mitchell et al. 2019).

Given the prevalence of hybridization in various taxa (occurring in 40% of plant families; (Whitney et al. 2010a) and the link between hybridization and diversification or radiation, a natural extension may be to ask if evolution in hybrids is more (or less) repeatable than evolution in nonhybrids. Do hybrids of similar genetic backgrounds but living in different environments tend to evolve in similar ways, or does spatiotemporal variation in selection and/or drift act on a large amount of genetic diversity in hybrids to generate divergent trajectories? On the one hand, hybrid evolution could be more constrained than nonhybrid evolution (Yeaman et al. 2018), as large numbers of alleles are often inherited together in nonrecombining chromosomal blocks. Furthermore, selection is likely to be stronger on hybrids than on nonhybrids since newly formed hybrids may be further from phenotypic optima, potentially also leading to more repeated evolution in hybrids vs. nonhybrids. Empirically, studies of hybrid speciation in sunflowers indicate that, at a coarse scale (chromosomal segments), the genomic composition of ancient spontaneous hybrids matches that of the subset of synthesized hybrids with high fitness (Rieseberg 2003), suggesting a degree of predictability or repeatability in hybrid evolution. Work on *Heliconius* butterflies found evidence for similar genetic and phenotypic changes across two different natural hybrid zones occurring between highland and lowland races, perhaps maintained via selection (Meier et al. 2021). Other studies suggest that repeated evolution of phenotype can result from selection acting upon diversity primarily generated by hybridization, such as flower color in monkeyflowers (Stankowski and Streisfeld 2015), plumage in wheatear birds (Schweizer et al. 2019), or in the radiation of cichlid fishes (Meier et al. 2017). On the other hand, evolutionary trajectories are sensitive to initial starting conditions (Arnold 1994; Donoghue 2005; Simões et al. 2008; Losos 2011), and hybrid evolution may be less repeatable since postF_1_ hybrids tend to have wide phenotypic variability, which can promote the evolution of new forms, as in adaptive radiation (e.g., Grant and Grant 2019). Phenotypic integration is often relaxed in hybrids, which can lead to novel trait combinations or transgressive phenotypes (trait values more extreme than those observed in either parent) (Stelkens and Seehausen 2009; Parsons et al. 2011; Pereira et al. 2014; Selz and Seehausen 2019). If different novel phenotypes confer benefits under different environments, then hybrids produced from the same parental taxa may demonstrate divergence across space rather than similar changes. For instance, radish hybrids derived from the same cross may start in different regions of phenotypic space depending on the environment and succeed in different environments by expressing different trait values (Hovick et al. 2012), which could lead to evolutionary divergence.

Here, we take an experimental approach to understanding the repeatability of hybridization using a synthetic hybrid and control lines of Texas sunflower (*Helianthus*). Previously, in a long‐term evolutionary experiment across eight generations, Mitchell et al. (2019) found that hybrids evolved significantly increased fitness, while nonhybrid controls did not, and that more traits evolved in hybrids than in controls. This previous work focused on insights from a single common garden to compare evolution in controls versus hybrids. However, this approach was not capable of drawing conclusions about the *repeatability* of evolution across multiple populations in different environmental contexts. To do so, we examine an additional set of hybrids and controls that evolved in a different location and add a second common garden site to assess repeated evolution in this system. Leveraging multiple generations, treatments, original planting “home sites”, and common gardens, we specifically ask the following questions: (1) Do hybrids consistently evolve more rapidly than controls (in terms of fitness and traits)?; (2) Do hybrids evolve in a more repeated manner than controls (in terms of overall phenotype and individual traits)?; and (3) Do hybrids repeat the evolutionary trajectory of a locally adapted phenotype?

## Materials and Methods

### STUDY SYSTEM

*H. annuus* ssp. *annuus* (L.) (the common sunflower) and *H. debilis* (Nutt.) (cucumberleaf sunflower) are annual sunflower species that hybridize in the wild where their ranges overlap (Heiser 1951). *H. a. annuus* in particular is known to hybridize widely with many annual sunflower species (Heiser 1951; Rieseberg 2003) and currently has a broad distribution, spanning most of the contiguous United States into Canada, although it is hypothesized to have had a narrower distribution in the Great Plains region before human contact (Whitney et al. 2010b). *H. debilis* has a much more restricted distribution and is found in areas along the Atlantic and Gulf coasts (Whitney et al. 2010b). *H. annuus* ssp. *texanus* is a morphologically distinct form of *H. annuus* that is found in central and southern Texas. It was once thought to be a hybrid lineage derived from *H. annuus* × *H. debilis*; putative introgression from *H. debilis* into *H. a. annuus* was thought to have allowed the southeastern expansion of *H. annuus* (sensu lato) into central and southern Texas (Heiser 1951, 1954; Rieseberg 2003). The latest data do not support that idea, indicating instead that the Texas form of *H. annuus* may have evolved from selection on standing variation within the species (Owens et al. 2021a). Here, we use *H. a. texanus* as a “locally adapted phenotype” of *H. annuus*, a reference point against which we can measure the evolution of phenotypes in our experimental hybrids and controls (see below).

### EXPERIMENTAL DESIGN

**Figure 1:**
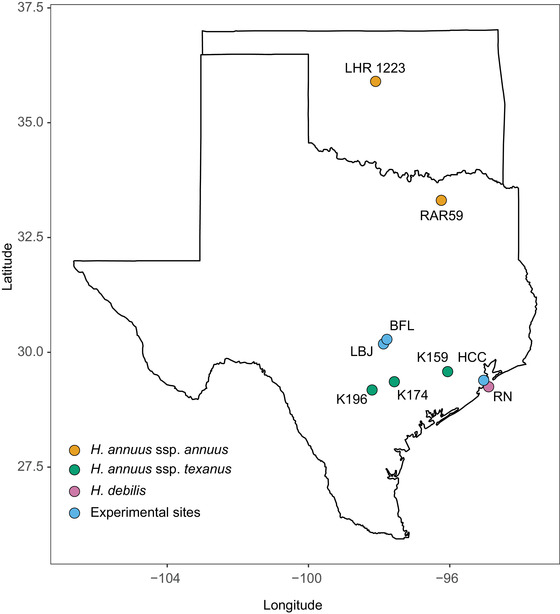
Locations for wild‐collected seed sources for *H. a. annuus* (gold), *H. a. texanus* (green), and *H. debilis* (pink), as well as the experimental home sites (blue). Plants from Oklahoma (LHR1223) were used to produce the F_1_ progeny and then backcrossed to the pollen donor from North Texas (RAR59) to produce the BC_1_ hybrids. RAR59 was also used to establish the controls. Home sites were established in 2003 at LBJ and BFL, and another was established at HCC in 2008. The final common gardens were planted at LBJ in 2017 and HCC in 2019.

We established controls and hybrids using wild‐collected seeds from *H. a. annuus* and *H. debilis*, as described in (Mitchell et al. 2019). We generated hybrids by crossing *H. debilis* from Texas with wild *H. a. annuus* from Oklahoma to produce F_1_ progeny and vegetatively propagated a single progeny from this F_1_ generation to produce F_1_ clones. *H. debilis* was used as the pollen donor to mimic the *H. annuus* cytoplasm predominantly found in *H. a. texanus*. We produced a BC_1_ line using a single *H. a. annuus* pollen donor from north Texas, producing individuals with approximately 75% *H. annuus* and 25% *H. debilis* genetic backgrounds. The limited number of parental individuals was a design choice to enable quantitative trait locus (QTL) analysis in this system (Whitney et al. 2015). We view this limited‐parent design as a conservative choice, as a mass‐crossing design would have produced larger amounts of genetic diversity in the hybrids and perhaps would have widened the gap in evolutionary responses between hybrids and controls. We used field‐collected seeds of *H. a. annuus* from this North Texas population as the controls (Fig. 1). *H. a. annuus* was chosen for the controls (rather than *H. debilis*), as this species represents the novel (colonizing) taxon with respect to the study area of interest, central and south Texas. Hereafter, we refer to the control and hybrid as “treatments”.

We germinated seeds for hybrids and controls, allowed them to establish, and then transplanted them (see Mitchell et al. (2019) for details) into one of three different “home sites”. In 2003, we established one pair of control and hybrid (BC_1_) populations at the Lady Bird Johnson Wildflower Center (LBJ, 30.18°N, 97.88°W) and a second hybrid population at the Brackenridge Field Laboratory (BFL, 30.28°N, −97.78°W) (Fig. 1). In 2008, we established another pair of control and hybrid populations (from the same control and hybrid seed stocks) at the Houston Coastal Center (HCC, 29.39°N, −95.04°W) (Fig. 1). Climate varies among the common gardens and seed collection sites, especially in terms of temperature and precipitation (Appendix S1, Table S1). All three home sites are located within the current range of *H. a. texanus*. We initiated each population using 500 individuals and allowed populations to evolve naturally at each home site, with annual disturbance via a rototiller (a machine used to till and aerate soil). We pulled wild sunflowers within an ∼250 m buffer surrounding each plot to minimize gene flow. Each year, we collected seeds from 96 individuals per population for use in the final common gardens. We stored seeds at 20°C in paper coin envelopes in plastic tubs filled with drierite (W.A. Hammond DRIERITE Co., Ohio, USA). We allowed plants to evolve through generation 8 at each of the three home sites.

### FINAL COMMON GARDENS

We germinated stored seeds from each generation, home site, and treatment, as well as seeds from wild‐collected *H. a. texanus*, at the University of New Mexico (UNM) (see Whitney et al. (2006) for a detailed germination protocol). Seedlings were then transported to Texas and transplanted. Originally, two common gardens were established in 2017, one at the LBJ home site and one at the HCC home site. We aimed to have 60 individuals per treatment × home site combination for starting, final, and wild *H. a. texanus* populations and 30 for intermediate generations, with intermediate generations only grown at the “home” site and final generations grown at both their “home” and “away” sites (BFL included as “away” at both final gardens). In 2017, not all intermediate generations were planted due to low germination rates (no individuals from generations 2–4 were included). In late August 2017, Hurricane Harvey destroyed much of the garden at HCC, leaving behind approximately 30 plants. Fortunately, we had a sufficient number of seeds saved and were able to redo the HCC common garden in 2019 using the same protocols. Hereafter, we refer to home sites by their location (LBJ, BFL, HCC) and common gardens by their year (2017, 2019). A visual schematic of the overall experimental design can be found in Figure S1 and compared to Figure S2, the experimental design associated with Mitchell et al. 2019.

Details for the 2017 common garden at LBJ are reported in Mitchell et al. (2019). For the 2019 common garden at HCC, we started germinating seeds at UNM in mid‐February and grew them in the greenhouse until late March, when they were transported to HCC and transplanted. We split individuals from each generation × treatment × home site evenly into two blocks (corresponding to the original locations within the site where the hybrids and controls were allowed to evolve) and randomized their location within the block. We transplanted a total of 1046 seedlings (523 per plot) over 3 days between March 30 and April 1, 2019. Seedlings were hand‐watered for the week following transplanting. Based on experiences in 2017 at HCC, we attempted to reduce plant predation by voles by mowing with a string trimmer between rows to prevent overwhelming growth of local vegetation that provides cover to the animals. We began mowing in early May and repeated it approximately every two weeks, with additional hand‐weeding of vining species.

### TRAIT MEASUREMENTS

We measured traits as described in Whitney et al. 2006, Whitney et al. 2010b *b)*; Mitchell et al. (2019). We measured 24 of the 27 traits measured in 2017 (Mitchell et al. 2019): specific leaf area (SLA), leaf dry matter content (LDMC), leaf succulence (Succ), leaf chlorophyll content (Chloro), leaf length to width ratio (LWR), leaf water use efficiency (WUE), bud initiation time (DaysToBud), seed maturation time (SMT), plant longevity, inflorescence disk diameter (DiskDiam), plant volume, height of lowest branch (HtLow), bushiness (Bushy), relative branch diameter (RelBrDiam), glandular trichome density (GlandDens), nonglandular trichome density (HairDens), damage by leaf‐vascular‐tissue feeders (SuckDam), damage by leaf chewers (ChewDam), damage by stem‐boring larvae (StemBorer), leaf carbon:nitrogen ratio (CNratio), stem or petiole weevil damage (WeevilDam), seed midge attack (MidgeDam), seed parasitoid attack (ParaDam), seed hole damage (HoleDam), seed gray seed weevil damage (GSW), and receptacle damage by larvae (RecepDam), excluding leaf longevity, bushiness, and gray seed weevil damage (Appendix S1 and Table S2).

### ANALYSES – INDIVIDUAL TRAITS

To estimate the evolution of fitness and individual traits, we ran individual Bayesian regression models for each common garden with standardized trait values as the responses and generation (time) as the predictor, with separate terms for controls and hybrids. These models were run for all combinations of trait × treatment for the home site plants at their respective common garden (i.e., LBJ home site plants at the 2017 LBJ common garden, HCC home site plants at the 2019 HCC common garden). We ran these from generation 1 through generation 8 (not generation 10) to make comparisons across common gardens. For the 2019 garden, we also include a random effect for block. Due to potential environmental gradients within blocks, we also added effects accounting for a plant's position (coordinates); however, models including these terms were not substantially better than the simpler models without coordinates according to DIC (deviance information criterion) comparisons, so we used the simpler models. Models were run using JAGS (Plummer 2003) through the R package R2jags (Su and Yajima 2009) using five chains, a burning of 2500 iterations, and a total of 75,000 iterations thinned every 25 iterations, resulting in 10,000 posterior samples. For all models, we checked MCMC convergence using traceplots and ensured that Rhat values were < 1.05. We deemed evolution in a given trait × treatment × home site × garden combination to be meaningful if the 95% credible interval for the estimated relationship between trait value and generation did not overlap zero.

To compare phenotypic evolution across sites, we ran two‐sided Pearson's correlation tests across pairwise comparisons using the slope of the regressions from the Bayesian analyses. Comparisons were made using estimates when grown at their home sites (e.g., LBJ in 2017, HCC in 2019) – we used the 2017 garden as the home site for BFL since it was geographically much closer than the 2019 garden. We performed these tests for both controls and hybrids to ask if they differed in the extent to which traits evolved similarly across sites.

In addition to running Bayesian linear models, we also ran individual linear models on 22 traits to test for repeated evolution (significant generation effect) and nonrepeated evolution (significant generation × home site interaction), as in Stuart et al. (2017). We ran these models separately for each garden and included individuals from all home sites. We used log‐transformed trait values as response variables and built models separately for controls vs. hybrids using the formula trait ∼ generation + home site + generation × home site. We extracted the partial associations (Eta‐squared) using the function *etasq()* in the package heplots in R (Friendly 2007) and then plotted the generation effect against the generation × home site interaction effect. We used a one‐way ANOVA to test for differences between hybrids and controls in trait deviation from the 1:1 line of the generation:generation × home site relationship.

### ANALYSES – MULTIVARIATE PHENOTYPE

To visualize and understand patterns of evolution of the different generation × treatment × home site combinations across multivariate phenotypic space, we performed phenotypic change vector analysis (PCVA) (Adams and Collyer 2009; Stuart et al. 2017). We log‐transformed and then standardized 22 traits (of the 24 that were measured at each common garden for a majority of plants, omitting traits from isotopic analysis that had limited sample sizes), including only complete observations, and ran principal component analyses on these traits using the *princomp()* function in R. We computed and mapped the centroids and standard error for the first three components for generation 1 and generation 8 hybrids and controls from each home site and the locally adapted *H. a. texanus*. We assessed (a) whether generation 8 hybrids and controls across home sites moved from generation 1 in the same or different directions along principal component axes and (b) whether generation 8 hybrids and controls moved from generation 1 toward or away from the locally adapted *H. a. texanus* along principal component axes. We then determined the number of principal component axes that captured 95% of the variation in each garden and computed centroids for each generation × treatment × home site combination as well as for *H. a. texanus*. We calculated the multidimensional Euclidean distance from each of these centroids to *H. a. texanus* to determine how far each treatment × home site combination was from the locally adapted phenotype and then determined whether they evolved toward or away from this phenotype by subtracting the generation 8 distance from the generation 1 distance. These calculations and comparisons were performed separately at each common garden.

We conducted a multivariate analysis of repeated evolution by estimating multidimensional vectors of divergence between generation one and generation eight at each home site for each treatment. We then measured differences in the angle (*θ_P_*) between vectors for pairwise comparisons within treatments using the code and functions of Stuart et al. (2017) as a reference. Briefly, we ran t tests comparing distributions of traits in generation one compared to those in generation eight for 22 traits that were measured at all sites and generations and used this t‐statistic to compute divergence vectors in 22‐dimensional phenotypic space. We calculated the angle (*θ_P_*) between these divergence vectors for every pairwise generation 1 – generation 8 comparison (separately at each common garden) by taking the dot product of each pair of divergence vectors, which was the arc‐cosine of the Pearson correlation for each vector pair.

In this context, a divergence angle (*θ*_P_) of 0° is perfectly “parallel” divergence (repeated evolution, or similar evolution across home sites), 90° is completely orthogonal divergence, and 180° is completely anti‐parallel (change in the opposite direction). To test for repeated evolution using these divergence angles, we tested whether the observed angles deviated significantly from orthogonal using a bootstrapping procedure, where divergence angles significantly lower than orthogonal are evidence for parallelism (Owens et al. 2021b). At each common garden, we resampled within each generation × treatment × home site combination with replacement, calculated divergence vectors between generation 1 and generation 8 as above, and then calculated *θ*_P._ We repeated this for 1000 bootstrap replicates to determine the proportion of bootstrap replicates that were 90 degrees or greater, multiplied by 2 (two‐tailed approach) (Stuart et al. 2017). To determine whether *θ*_P_ differed significantly between control versus hybrids, we used output from the above bootstrap procedure and subtracted the LBJ – HCC hybrid *θ*_P_ from the LBJ – HCC control *θ*_P_ to determine the proportion of bootstrap replicates for which the hybrid *θ*_P_ was greater than the control *θ*_P_, multiplied by 2 (conservative two‐tailed approach).

### ANALYSES – PREDICTING THE SPEED OF EVOLUTION

We calculated the distance (for each trait) from the mean value of the initial (2003) generation hybrids and controls to that of *H. a. texanus* (the locally adapted phenotype). Across all traits, we estimated the correlation between this distance and the slope from the Bayesian analysis (as a measure of the speed of evolution) across all traits using the *lm*() function in R, as in (Mitchell et al. 2019).

## Results

### EVOLUTION OF FITNESS AND TRAITS IN HYBRIDS VERSUS CONTROLS

**Figure 2:**
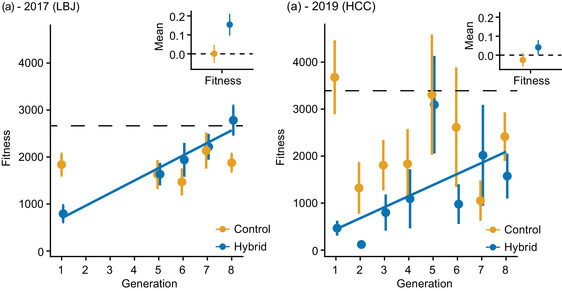
Fitness evolves in hybrids across locations, more so at LBJ. Data are raw fitness means (seed set) and standard errors from LBJ plants grown at LBJ garden (a) and HCC plants grown at HCC garden (b), control in orange, hybrid in blue. The dashed line indicates the mean fitness of *H. a. texanus* grown in that common garden. Insets: mean slope estimate and 95% credible intervals from Bayesian fitness models. Panel (a) redrawn from Mitchell et al. (2019).

Across home sites, hybrids evolved significantly higher fitness over time, while controls did not, and the magnitude of fitness increase was greater for LBJ hybrids than HCC hybrids (Fig. 2). In the 2017 common garden, the LBJ‐grown hybrids had an estimated slope of 0.154 [0.096, 0.211] (posterior mean and 95% credible interval), and the controls had a slope of 0.001 [−0.047, 0.047]. Likewise, in the 2019 common garden, the HCC‐grown hybrids had a slope of 0.041 [0.003, 0.079], and the controls had a slope of −0.025 [−0.062, 0.011]. This latter relationship in the controls was significant (but negative) at the 80% credible level. Notably, overall fitness was higher in the 2019 garden at HCC, and fitness increased from a very low initial value to reach the mean *H. a. texanus* fitness value in both gardens (Fig. 2).

**Figure 3:**
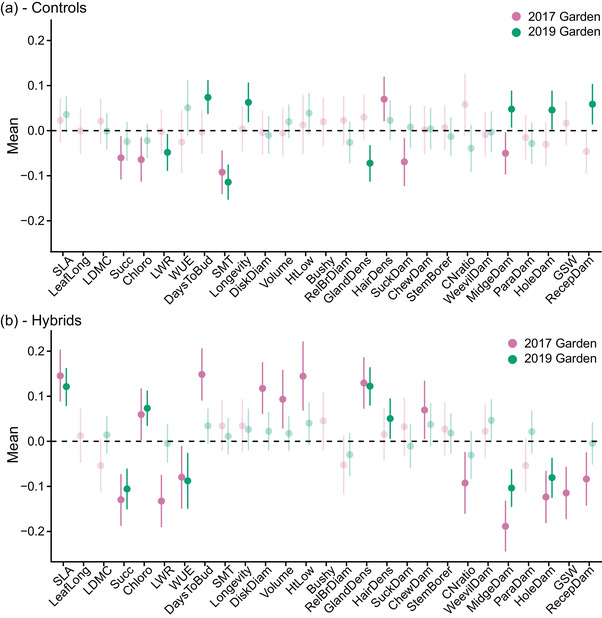
Evolution of traits across common gardens for controls (a) and hybrids (b). Points are the mean slope value from the Bayesian regressions of trait value on generation with 95% credible intervals represented by lines. LBJ home site plants grown at the 2017 garden at LBJ are in pink, and HCC home site plants grown at the 2019 garden at HCC are in green. Dark points and lines are significant at the 95% credible level, while transparent points and lines are not. See Table S3 for the full results, including BFL.

More traits evolved significantly in hybrids compared to the controls at the 2017 garden, but the number of traits that evolved did not differ between treatments at the 2019 garden (Table S3, Fig. 3). At the 2017 garden, 16 of the 27 measured traits (59%) evolved significantly in LBJ hybrids, compared to six out of 27 (22%) in the LBJ controls (*Χ* ^2^ = 4.55, *df* = 1, *p* = 0.033). At the 2019 garden, eight of the 24 measured traits (33%) evolved in HCC hybrids, while eight out of 24 (33%) also evolved in the HCC controls (*Χ* ^2^ = 0, *df* = 1, *p* = 1). Recall that three traits from the 2017 common garden were not measured in 2019 (see Materials and Methods above). See Table S3 for the evolution of traits of all treatment × home site combinations, including BFL.

### REPEATED EVOLUTION OF INDIVIDUAL TRAITS IN HYBRIDS VERSUS CONTROLS

**Figure 4:**
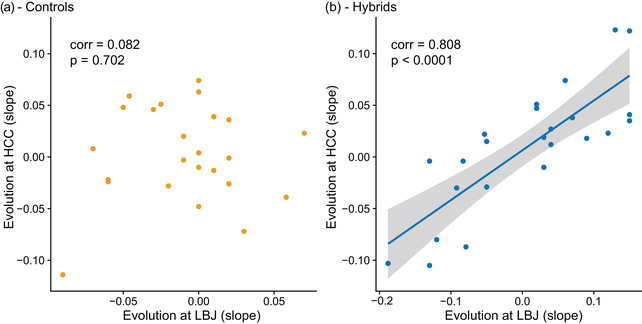
Trait evolution is correlated across sites in hybrids but not in controls. Points represent evolution (as measured by slope obtained from the Bayesian regression analyses) at each home site: (a) control LBJ plants grown at LBJ (2017) versus control HCC plants grown at HCC (2019); (b) hybrid LBJ plants grown at LBJ (2017) versus hybrid HCC plants grown at HCC (2019). The line is the result of a loess smoothing function, with a 95% confidence interval depicted in gray shading. Correlations and p values from Pearson's two‐sided test.

Hybrid trait evolution was correlated across home sites in all pairwise comparisons, while this was not the case for controls (Fig. 4). Control evolution at LBJ vs control evolution at HCC, as measured by the slopes obtained from the Bayesian regressions for each trait, was not correlated (Fig. 4a, *r* = 0.082, *p* = 0.702). The evolution of hybrid traits between LBJ and HCC, LBJ and BFL, and HCC and BFL were all correlated (*r* = 0.808, *p* < 0.001; *r* = 0.763, *p* < 0.001, *r* = 0.771, *p* < 0.001, Fig. 4b).

**Figure 5:**
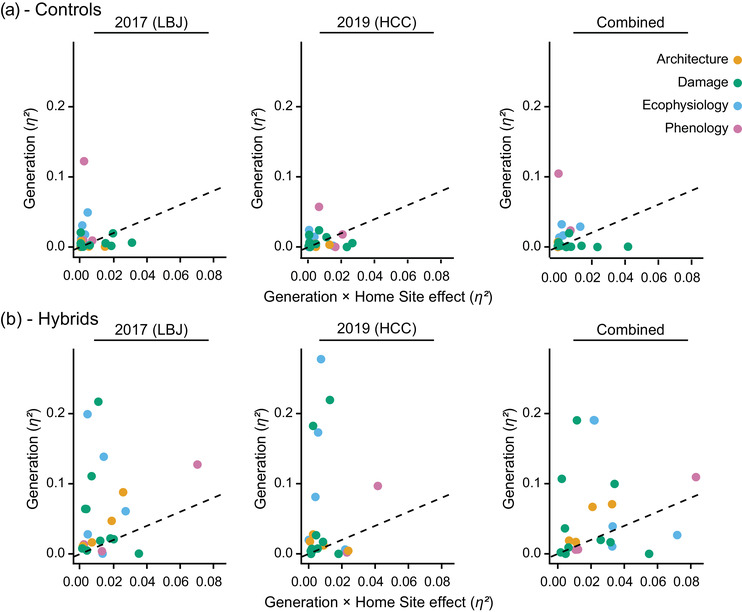
Repeated evolution in individual phenotypic traits. Separate linear models were built for each log‐transformed, standardized trait to estimate effect sizes for the generation effect (a measure of repeated evolution across sites) and generation × home site effect (a measure of the extent to which divergence is dependent upon where plants evolved) separately for controls (a): at LBJ in 2017, 6/22 (27%) with a significant generation effect; at HCC in 2019, four of 22 (18%) with a significant generation effect; and at both their respective home sites, 5/22 (23%) with a significant generation effect, and hybrids (b): at LBJ in 2017, 14 of 22 (64%) with a significant generation effect; at HCC in 2019, 12 of 22 (55%) with a significant generation effect; and at both their respective home sites, 15 of 22 (68%) with a significant generation effect. Generation 1 plants were reused for BFL, HCC, and LBJ home sites at that common garden to estimate changes for each home site from the same initial starting population. The dashed line indicates the 1:1 line – values above this line indicate a large effect of repeated evolution. Each point represents a single trait and is colored by trait category (orange = Architecture, green = Damage, blue = Ecophysiology, pink = phenology). See Table S4 for modeling results.

We also measured the repeated evolution of phenotypic traits in a different way by building linear models and comparing effect sizes for the generation × home site effect vs the generation effect alone. The effect of generation alone is a measure of repeated evolution across sites, while the generation × home site effect measures the extent to which divergence is dependent upon where plants evolved (deviation from repeated evolution). Using this approach, we also found a large effect of repeated evolution in hybrids when compared to controls (Fig. 5, Table S4). In the controls at LBJ in 2017, 6/22 traits (27%) had a significant generation effect, while at HCC in 2019, 4/22 traits (18%) had a significant generation effect (Fig. 5a). Comparing both at their respective home sites, 5/22 traits (23%) had a significant generation effect. In hybrids at LBJ in 2017, 14/22 traits (64%) had a significant generation effect, while at HCC in 2019, 12/22 traits (55%) did (Fig. 5b). Comparing both at their respective home sites, 15/22 traits (68%) had a significant generation effect. Moreover, in hybrids, the relationship between the generation effect and the generation × home site effect was above the 1:1 line for more traits and further above that line, indicating potentially greater evidence for repeated (versus nonrepeated) divergence in hybrids. Compared to controls, hybrids deviated in a more positive manner from the 1:1 line at both common gardens (ANOVA), although this was not statistically significant when analyzing plants at their home site (LBJ: *F* = 5.31, *df* = 1, *p* = 0.026; HCC: *F* = 5.18, *df* = 1, *p* = 0.028; combined *F* = 2.96, *df* = 1, *p* = 0.093) (Fig. 5, Table S4).

### REPEATED EVOLUTION OF OVERALL PHENOTYPE IN HYBRIDS VERSUS CONTROLS

**Figure 6:**
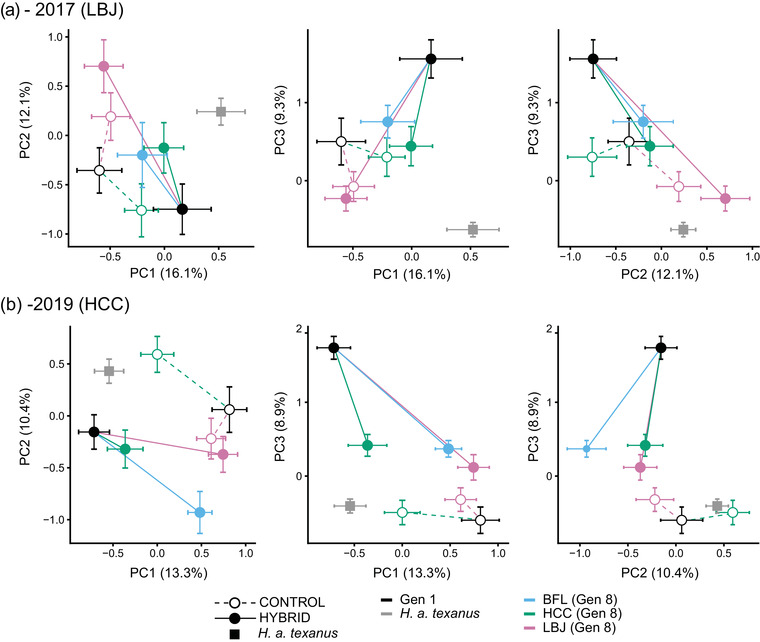
The multivariate phenotype in hybrids tended to evolve in a repeated manner across sites, while controls did not at (a) the LBJ garden in 2017 and (b) the HCC garden in 2019. Here, each point represents the centroid from a PCA for a particular generation × treatment × home site. Lines connect generation 1 (black) to generation 8 from different home sites (BFL = light blue, HCC = green, LBJ = pink); dashed lines and open circles are controls, solid lines and filled circles are hybrids. *H. a. texanus* is in gray square. Error bars represent standard errors.

Multivariate phenotypes in hybrids tended to evolve in similar ways across sites, while controls did not. We used principal component analysis to reduce the number of dimensions across traits separately at each common garden. In the 2017 common garden, the first 16 components described 95% of the variation (Table S5). In the 2019 common garden, the first 17 components described 95% of the variation (Table S5). We examined phenotypic space in the first three components separately for each garden and found that hybrids evolved from their starting point to similar points in trait space across the home sites, while controls did not (Fig. 6).

**Figure 7:**
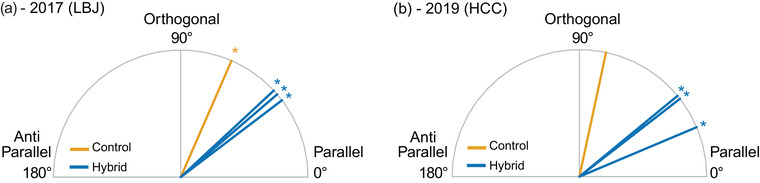
Tests of phenotypic parallel divergence. Values for differences in pairwise angles in degrees (*θ*_P_) at (a) the 2017 LBJ common garden and (b) the 2019 HCC common garden. The control comparison (LBJ control vs. HCC control) is in gold, and hybrid comparisons (the three possible pairwise comparisons between LBJ, HCC, and BFL hybrids) are in blue. Asterisks indicate that the angle is significantly different from orthogonal (90°) based on 1000 bootstrap resamples with replacement. See Table S6 for full results.

We used the PCVA framework to explicitly test differences in the angle (*θ*_P_) of the multidimensional trait vectors between home sites using permutation tests. If the divergence angle is significantly less than 90°, we interpret this as evidence for repeated evolution across sites. In 2017, the hybrids exhibited greater parallelism than controls (Table S6, Fig. 7a), where the hybrid *θ*_P_ LBJ – HCC comparison was 37.1°, LBJ – BFL was 40.9°, and HCC – BFL was 43.0° compared to the control *θ*_P_ LBJ – HCC of 66.3°. Additionally, using a conservative two‐tailed approach on the permuted data to compare the hybrid angle with the control angle, the LBJ – HCC hybrid *θ*_P_ was marginally greater than the LBJ – HCC control *θ*_P_ (*p* = 0.062). For all hybrid and control comparisons in 2017, the divergence angles *θ*_P_ were significantly less than 90° based on the bootstrap resampling procedure (*p* < 0.001). In 2019, the hybrids also exhibited greater repeatability than controls (Table S6, Fig. 7b), where the hybrid *θ*_P_ LBJ – HCC comparison was 39.4°, LBJ – BFL was 22.8°, and HCC – BFL was 38.1° compared to the control *θ*_P_ LBJ – HCC of 78.0°. Using the two‐tailed approach to compare the hybrid and control angles, the LBJ – HCC hybrid *θ*_P_ was significantly greater than the LBJ – HCC control *θ*_P_ (*p* = 0.018). For all hybrid comparisons in 2019, the divergence angles *θ*_P_ were significantly less than 90° based on the bootstrap resampling procedure (*p* < 0.001), while the control comparison was marginally less than 90° (*p* = 0.074). Across both gardens, all but one hybrid‐control comparison were not less than 90°, indicating little evidence for repeated evolution when comparing evolution between hybrids and controls (Table S6).

### EVOLUTION TOWARD THE PHENOTYPE OF THE LOCALLY ADAPTED *H. a. texanus*

Both hybrids and controls tended to evolve toward the phenotype of the locally adapted *H. a. texanus* along at least some principal component axes (Fig. 6, locally adapted phenotype depicted in gray). If we incorporate the first *n* principal component axes that account for 95% of the variation (16 axes in 2017 and 17 axes in 2019), in both home sites, the hybrid starts further away from *H. a. texanus* than the control does (generation 1), but by generation 8, the controls and hybrids are similar distances away (Table 1). Although both controls and hybrids moved toward the phenotype of the locally adapted taxon across generations, the magnitude of that change was greater in hybrids, indicating that hybrids evolved more rapidly toward the phenotype of *H. a. texanus* (Table 1).

**Table 1:** Euclidean distances between experimental controls and hybrids and the locally adapted *H. a. texanus* in multivariate phenotype space. Distances are computed using the centroids of the principal components that account for 95% of the variation in each garden (16 PCs in 2017 and 17 PCs in 2019) for each generation × treatment × home site combination and the locally adapted taxon. Changes in distance are calculated by subtracting the Generation 8 distance from the Generation 1 distance for each treatment × home site combination, where negative values indicate that the distance to *H. a. texanus* has decreased over generations

		Distance from *H. a. texanus*				Change in Distance		
Garden	Treatment	Gen1	HCC (Gen 8)	LBJ (Gen 8)	BFL (Gen 8)	HCC	LBJ	BFL
2017 (LBJ)	Control	2.458	1.967	1.661		−0.491	−0.797	
	Hybrid	3.141	1.734	1.705	2.112	−1.407	−1.436	−1.029
2019 (HCC)	Control	2.157	1.531	1.909		−0.626	−0.248	
	Hybrid	2.813	1.640	1.940	2.212	−1.174	−0.873	−0.601

**Figure 8:**
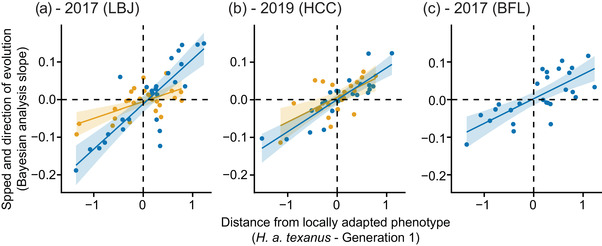
Distance from the locally adapted phenotype predicts trait evolution. (a) LBJ in 2017; (b) HCC in 2019; (c) BFL in 2017. For both controls (orange) and hybrids (blue), the rates of evolution for individual traits (slopes obtained from the Bayesian evolution analyses) are significantly related to the distance from the initial mean trait value (BC_1_ and initial *H. a. annuus* generation for hybrids and controls, respectively) to the mean trait value of *H. a. texanus*. Each point represents an individual trait, lines represent loess smoothed relationships, and shading represents the 95% confidence intervals of the smoothed lines. Traits were standardized across all plants analyzed prior to comparisons. (a) Control: coefficient = 0.044, *p* = 0.001, Adj‐*R* ^2^ = 0.365, Hybrid: coefficient = 0.120, *p* < 0.001, Adj‐*R* ^2^ = 0.683); (b) Control: coefficient = 0.065, *p* = 0.002, Adj‐*R* ^2^ = 0.358; Hybrid: coefficient = 0.085, *p* < 0.001, Adj‐*R* ^2^ = 0.738; (c) Hybrid: coefficient = 0.065, *p* < 0.001, Adj‐*R* ^2^ = 0.506; linear regression. Panel (a) is redrawn from Mitchell et al. (2019).

We also asked whether the distance from the locally adapted *H. a. texanus* phenotype of individual traits predicted the speed and direction of evolution in both hybrids and controls. In every scenario, the distance from the initial standardized mean trait values of our experimental treatments (BC_1_ or control generation 1) to the *H. a. texanus* mean trait value was significantly positively related to the speed and direction of evolution (slope from our Bayesian analyses). For example, if a *H. a. texanus* trait value was higher than the initial value in an experimental population and far away, the trait in the experimental group tended to evolve quickly and toward a higher value. We previously found this at the LBJ garden (Mitchell et al. 2019; Fig. 8a), where the hybrid coefficient was 0.120 (*p* < 0.001, Adj‐*R* ^2^ = 0.683) and the control was 0.044 (*p* = 0.001, Adj‐*R* ^2^ = 0.365); in the current study, we found the same pattern at HCC in 2019 (hybrid coefficient = 0.085, *p* < 0.001, Adj‐*R* ^2^ = 0.738; control coefficient = 0.065, *p* = 0.001, Adj‐*R* ^2^ = 0.358; Fig. 8b) and for the BFL hybrids in 2017 (coefficient = 0.065, *p* < 0.001, Adj‐*R* ^2^ = 0.506; Fig. 8c). Note that these relationships were slightly stronger for hybrids but still significant for controls, and overall distance from *H. a. texanus* explained a substantial proportion of the variation in the magnitude of trait evolution (moderately high adjusted‐*R* ^2^ values).

## Discussion

We found that hybrids consistently evolved higher fitness across environments and that overall hybrid phenotypes evolved in a more repeatable way than controls did. Furthermore, resynthesized hybrids (to some extent) evolved toward the phenotype of a historical, locally adapted taxon, perhaps indicative of repeated evolution across deeper timescales as well. We observed a continuum of evolutionary responses, and differences in the degree of repeatability of evolution in specific traits may indicate the role of selection by potential environmental drivers.

### EVOLUTION IN HYBRIDS VERSUS NON‐HYBRIDS

Overall, fitness showed consistently greater evolutionary changes in hybrids than controls (Fig. 2). This work builds on previous results examining the LBJ and BFL lines at a single common garden (Mitchell et al. 2019) but with replication (the HCC lines) that allows for a more nuanced understanding of the evolution of hybrids versus controls. These findings are in line with the idea that lineages can increase fitness via the incorporation of advantageous alleles from other species, i.e., adaptive introgression (Rieseberg and Wendel 1993; Hedrick 2013; Suarez‐Gonzalez et al. 2018 p.). Given that hybrids consistently had very low starting fitnesses across home sites, it is unsurprising that they evolved higher fitness within just a few generations, although predictable increases in fitness can be driven by different genetic (and phenotypic) trajectories (Simões et al. 2008; Kryazhimskiy et al. 2014). Our results are most applicable to hybrid populations rather than rare hybridization events in otherwise homogeneous populations. In such cases, we might expect much more stochastic loss of admixed alleles leading to less repeatability.

There was less consistency in the evolution of individual phenotypic traits, where more hybrid traits (compared to controls) evolved at one home site but not at the other (Figs. 2 and 3, Table S3). This discrepancy is driven both by the evolution of more traits in the controls at HCC (compared to LBJ) and the evolution of fewer traits in the HCC hybrids. We discuss potential reasons for the differences between home sites further below.

### REPEATABLE HYBRID EVOLUTION

Replicated experiments have provided some of the best, most comprehensive evidence for repeated evolution, as in the evolution of Trinidadian guppies in response to different predator environments (reviewed in Reznick and Ghalambor 2005). Naturally, replicated “experiments,” such as in freshwater stickleback fish or multiple hybrid zones of *Heliconius*, also demonstrate some evidence for parallelism at the genetic and phenotypic levels (Kingman et al. 2021; Meier et al. 2021). Here, we leveraged replicated hybrid and control population pairs and reciprocal common gardens and found that hybrid traits tended to evolve in similar ways across sites (Figs. 3 and 4, Table S3) and that they evolved in a more repeatable way than controls did (Figs. 5 and 6, Table S4).

The likelihood of repeated evolution increases in concert with two quantities, both of which likely differ between hybrids and nonhybrids: the strength of selection and the degree of genetic constraint on evolution (Yeaman et al. 2018). When evolution occurs due to novel mutations, the likelihood of repeated evolution increases as the number of possible beneficial mutations decreases (Orr 2005). When selection acts instead on standing genetic variation, the likelihood of repeated evolution increases with the strength of selection and is more likely to occur in genes with large phenotypic effects (MacPherson and Nuismer 2017). In hybrids, the units of selection are often large chromosomal blocks composed of hundreds or thousands of genes; therefore, hybrid evolution should be more constrained than controls, consistent with the evidence presented here. In *Helianthus*, nonrecombining haploblocks are responsible for some ecotypic differences within species and may be due to introgression from other species (Todesco et al. 2020). This is further supported by evidence that signals of repeated evolution in natural *Helianthus* populations are disproportionately caused by haploblocks (Todesco et al. 2020; Huang 2021). Moreover, selection is likely to be stronger on hybrids than on nonhybrids since newly formed hybrids may be further from phenotypic optima (as in our case, where hybrids experienced a greater change in the direction of the locally adapted phenotype of *H. a. texanus* than controls, Table 1).

We compared the extent of repeatability under two scenarios defined by Stern as “collateral evolution,” where repeated evolution in our controls represents evolution from standing genetic variation (scenario 1) and repeated evolution in our experimentally generated hybrids represents evolution via the introduction of alleles from outside populations (scenario 2) (Stern 2013). Although there is evidence for collateral evolution via hybridization in other systems (e.g., Stankowski and Streisfeld 2015; Meier et al. 2017; Schweizer et al. 2019), we believe that this is the first study to investigate this question in both an experimental context and in a field setting (Mitchell and Whitney 2018). Although our focal sites have different environmental contexts, especially in terms of precipitation (Table S1), they may represent similarly novel environments when compared to the historical range of *H. a. annuus* before human colonization of North America (Whitney et al. 2010b) and thus may create similar selective pressures. We also note that controls were *H. a. annuus* sourced from north Texas to represent the majority background of *H. a. texanus*; if we had chosen *H. debilis* as controls (sourced from closer to the Gulf Coast), we may have seen different patterns across environments associated with local adaptation.

Other studies have investigated the role of additional diversity‐generating or diversity‐maintaining processes on the repeatability of evolution. For instance, sexual (versus asexual) reproduction had a complex effect on repeatability in *Chlamydomonas* algae, where the effects varied depending on the initial genomic composition and environments of starting populations (Lachapelle and Colegrave 2017), although sex overall sped up adaptation in terms of fitness in yeast (McDonald et al. 2016), and recombination similarly sped up adaptation in *E. coli* (Cooper 2007). Polyploidy is another diversity‐generating mechanism, and some aspects of polyploidization are also repeatable (reviewed in (Rieseberg 2001; Soltis and Soltis 2009)).

In contrast to adaptive explanations, one factor contributing to repeated evolution in our hybrids could be the purging of incompatible introgressed ancestry. There is significant pollen and seed sterility in F_1_ hybrids between *H. annuus* and *H. debilis* (Heiser 1951; Chandler et al. 1986; Lai et al. 2005). Both could drive repeated genomic and phenotypic evolution without local adaptation. In this experiment, we only measured female fitness, but seed fertility is strongly correlated with pollen viability, so we likely indirectly measured the latter as well (Rieseberg 2000).

### NONREPEATABLE EVOLUTION IN HYBRIDS

Overall, our findings spanned the range of evolutionary responses in terms of repeatability. Differences in the number of traits in hybrids that evolved across home sites (16 at LBJ and 8 at HCC at the 95% credible level) (Fig. 3, Table S3) indicate that hybrid evolution in our experiment also occurred in nonrepeated ways across sites in Texas. Our analyses recapitulate the work of Stuart et al. (2017) examining lake‐stream population pairs of stickleback fish evolution, in which a continuum of evolution in terms of parallelism and environmental dependence was found. Similarly, in a meta‐analysis of parallel evolution across fish species, cases of nonparallel evolution across habitat boundaries were more common than cases of parallel evolution (Oke et al. 2017). Overall, there has been a shift toward viewing parallel versus nonparallel evolution as a continuum, rather than binary outcomes, with differences in evolutionary responses among replicates or traits even when exposed to similar pressures (reviewed in Bolnick et al. 2018).

Due to the long‐term, experimental nature of our study, we lack the replication necessary to link differences to specific environmental variables; however, we can hypothesize reasons underlying the differences in evolution at the three sites. The LBJ and BFL sites are both located in Austin, Texas, although they are found in very different habitats (clay soil in oak savanna versus sandy river bottom soil, respectively). In contrast, the HCC site is located south of Houston, Texas, in very heavy clay soil, approximately 300 km southeast of Austin (Fig. 1). Thus, LBJ and BFL are geographically closer to the core range of *H. a. annuus*, while HCC is both geographically and environmentally more distinct (Table S1). Since controls were collected from a site located in northern Texas, the HCC site may have been far enough outside the “locally adapted” environment for the *H. a. annuus* parental species that novel selective pressures acted on both the control and hybrids, while for the Austin sites, these pressures were not as extreme.

A lack of repeatability in specific traits (or genetic changes) is not unexpected, while fitness evolution is more predictable (Kryazhimskiy et al. 2014). A laboratory experimental study on *Drosophila* found that the outcome of fitness evolution was less dependent on initial conditions than were the outcomes for individual traits (Simões et al. 2008). Moreover, in long‐term studies in the *Drosophila* system, there was substantial variation in outcome depending upon the length of the study, and despite some broad patterns of convergence, differentiation in certain traits among populations persisted in the long term across 60 generations compared to our eight generations (Simões et al. 2019). Population size may also play a role in the repeatability of evolution, where genetic drift can decrease the likelihood of parallel changes (Szendro et al. 2013). Our initial starting population size of 500 may be large compared to the sizes of initial hybrid populations formed in nature. Population size was influential in experimental work in algal populations in response to high salt environments, where adaptation (as measured by growth rate) to the novel environment was not repeatable in small populations, and adaptation was more repeatable in medium or large‐sized populations (Lachapelle et al. 2015).

### EVOLUTION TOWARD THE LOCALLY ADAPTED PHENOTYPE

Overall, we found that both controls and hybrids tended to evolve toward the locally adapted phenotype of *H. a. texanus*, but hybrids did this to a greater extent (Table 1, Fig. 6). Our use of resynthesized hybrids was inspired by the work of (Rieseberg 2003), which found that the genomic composition of a subset of resynthesized hybrids (those with high fitness) between annual species of *Helianthus* matched the genomic composition of ancient, natural hybrids, suggesting that hybrid evolution can be replayed to some extent. Other work along these lines has documented different degrees of repeated evolution toward historically important values. For instance, in *Drosophila subobscura*, populations sampled from different geographic areas did not converge in a new environment in a laboratory setting, but one population did converge toward an “older” laboratory population from the same natural location, suggesting that historical context (perhaps standing genetic variation) was important (Seabra et al. 2018). In another *Drosophila* example, experimental “reverse” evolution found evidence for convergence toward ancestral levels of adaptation (Teotónio et al. 2009). Evolution may thus be (partially) replayed using experimental work.

Additionally, the degree of phenotypic “mismatch” (for a given trait, the distance between the initial generation's value and the trait value of the locally adapted *H. a. texanus*) predicted the rate of evolution (Fig. 8). We reported this finding for LBJ and BFL plants in previous work (Mitchell et al. 2019), and here we confirm that this pattern exists at our third, more environmentally distinct site, although the difference in the strength of the relationship (stronger in hybrids than controls) is more notable at LBJ than at HCC. For both hybrids and controls, we thus have some evidence for evolution “replaying” itself, which was slightly stronger for hybrids. A similar pattern was observed at the genomic level in stickleback fish, where the best predictor for the speed of evolution in the transition to freshwater environments was the amount of ecotypic differentiation between marine and long‐established freshwater populations (Kingman et al. 2021).

Evolution in the locally adapted *H. a. texanus* could be due to standing genetic variation (rather than variation arising due to hybridization) associated with segregating haploblocks formed by inversions, which contribute to differentiation in this system as well as *Helianthus* more broadly (Owens et al. 2021a; Todesco et al. 2020). This standing genetic variation hypothesis could also explain some of the convergence observed between the control populations and *H. a. texanus* (Fig. 6, Table S6). Additional work is necessary to understand the potential structural genomic contributions to evolution in this experiment.

### CONCLUSIONS AND FUTURE DIRECTIONS

Here, we use a replicated evolutionary experiment in a natural field setting to understand the strength and repeatability of hybrid evolution, both across environments and relative to a locally adapted phenotype. We demonstrate that (1) hybrids consistently demonstrate more rapid fitness evolution than controls; (2) individual traits sometimes, but not always, evolve more rapidly in hybrids than controls; (3) hybrid phenotypic evolution is predictable across sites and generally occurs more in a more repeatable manner than does control phenotypic evolution; and finally, (4) both hybrids and controls demonstrate some “replaying” of evolution toward the locally adapted phenotype. Future work is needed to understand the role of haploblocks in the evolution of our resynthesized hybrids and whether genetic changes have also occurred in a repeated fashion. Additional replication is necessary to determine the extent to which similarities or differences in environmental selective pressures (climate, soil, microbial community, etc.) determine repeated versus nonrepeated evolution. Moreover, comparisons between repeated elements (genetic or phenotypic) in our *Helianthus* example might be compared at a macroevolutionary scale with other plant examples, for instance, asking whether the same traits evolve similarly in distantly related plant groups, as there is some exciting evidence for the involvement of similar genetic changes across well‐studied vertebrate examples of repeated evolution (Kingman et al. 2021). Finally, with increased rates of hybridization related to human‐induced global change and movement of species, it is important to understand the potential fates of hybridizing populations in terms of conservation and global biodiversity issues (Guo 2014).

## AUTHOR CONTRIBUTIONS

K.D.W., L.H.R., N.M., and G.L.O. conceived of the study. N.M. and H.L. carried out the study and collected data. N.M. performed the analyses. N.M. and K.D.W. wrote the initial manuscript. All authors contributed to review and editing.

## CONFLICT OF INTEREST

The authors declare no conflicts of interest.

Associate Editor: J.W. Busch

Handling Editor: A.G. McAdam

## Supporting information


**Figure S1**. Experimental design for the current study.
**Figure S2**. Experimental design for the 2017 common garden as reported in Mitchell et al. 2019.
**Table S1**. Climatic characterization of collection sites and common gardens.
**Table S2**. Fitness and 24 traits measured across both final common gardens.
**Table S5**. Principal component information for morphological analyses.
**Table S5**. Principal component information for morphological analyses.
**Table S5**. Principal component information for morphological analyses.
**Table S6**. Tests of phenotypic parallel divergence.

## Data Availability

All relevant data and code generated for this work are available via the Dryad Digital Repository (https://doi.org/10.5061/dryad.zgmsbccdq).
