# Supplementary material for: Hybrid evolution repeats itself across environmental contexts in Texas sunflowers (Helianthus)
Source: Evolution. 2022 Jun 17;76(7):1512–28. doi: 10.1111/evo.14536 (PMC9544064; doi:10.1111/evo.14536)
Supplement: Supplementary file 1 — Figure S1. Experimental design for the current study. Figure S2. Experimental design for the 2017 common garden as reported in Mitchell et al. 2019. Table S1. Climatic characterization of collection sites and common gardens. Table S2. Fitness and 24 traits measured across both final common gardens. Table S5. Principal component information for morphological analyses. Table S5. Principal component information for morphological analyses. Table S5. Principal component information for morphological analyses. Table S6. Tests of phenotypic parallel divergence. [file EVO-76-1512-s001.docx]

**SUPPORTING INFORMATION**

**Hybrid evolution repeats itself across environmental contexts in Texas sunflowers (*Helianthus*)**

**Appendix S1**

**Characterization of climate**

To characterize the climate, we downloaded bioclimatic variables for each of our collection sites and common gardens from WorldClim (Fick and Hijmans 2017) at the 0.5-minute scale using the *getData()* function in the R package raster (Hijmans and van Etten 2014). Data are reported in Table. S1.

**Trait measurements**

We measured traits as described in Whitney et al. (2006), Whitney et al. (2010) and Mitchell et al. (2019), see these references for additional details and Table S2 for a list of all 24 traits and their units measured at both common gardens.

For leaf/ecophysiological traits, two recently fully expanded leaves per plant were selected before flowering began to estimate leaf traits. For one leaf, chlorophyll content was estimated and averaged across five sections of the leaf using a SPAD meter (Spectrum Technologies, Aurora, IL). For the same leaf, fresh mass was measured on a microbalance, leaves were scanned on a flatbed scanner for estimation of area, length, and width using ImageJ, and leaves were then dried in a drying oven until constant mass was reached and dry mass was measured. Values of specific leaf area (SLA, area / dry mass), Leaf succulence (Succ, fresh mass – dry mass / area), leaf dry matter content (LDMC, dry mass / fresh mass), and leaf length to width ratio (LWR, length/width) were computed using these measurements. Leaf disks (taken using a #7 cork borer) were taken and dried from the second leaf, ca. 3mg were weighed in tin and analyzed for Carbon, Nitrogen, ^13^C, and ^15^N content at the University of New Mexico Center for Stable Isotopes to estimate leaf Carbon:Nitrogen ratio (CNratio) and water use efficiency (WUE, δ^13^C). Only a subset of individuals was analyzed for leaf nutrients (30 per lineage for each generation 1 and final generation lineage). A second leaf disk was used to measure the densities of glandular and nonglandular trichomes (GlandDens, HairDens) on the abaxial leaf side. Trichomes were counted under 5× magnification with a 1cm × 1cm reticle (0.2cm × 0.2cm, 0.04cm^2^ area) and converted to densities.

Phenological status of all plants was assessed every third day from early May until early November. From these data, we calculated bud initiation time (DaysToBud) as the number of days between initial transplantation and appearance of immature apical flowering head, seed maturation time (SMT) as number of days between end of female receptivity and achene maturity, plant longevity as the number of days between transplantation and mortality.

For plant architectural traits, disk diameter (DiskDiam) was measured as the diameter of the central disk of the apical flowering head during female receptivity. After mortality, the height of the lowest branch (HtLow), relative branch diameter (RelBrDiam) (average branch diameter across all primary branches with diameters > 3mm divided by basal stem diameter), and plant volume (*V* = π × *r*2 × *l*, where *r* is half the basal stem diameter *l* is the height of the plant) were measured.

Throughout the season, seedheads were enclosed in mesh bags (DelStar Technologies, Delaware) after pollination and before seed drop with a goal of at least four seedheads per plant. After mortality, all seedheads (bagged and unbagged) were counted and collected. The number of viable seeds per seedhead by through visual inspection and counting. Fitness (viable seed production for each plant) was estimated by multiplying the total number of heads produced by the average number of viable seeds per head. Damage to seedhead receptacles by Lepidoptera was measured by counting the number of larval holes and estimating the average per head (RecepDam). Seed damage was also visually assessed using a dissecting microscope (Leica, Wetzlar, Germany), including the number subject to parasitoid attack (ParaDam), damage by the sunflower midge *Neolasioptera helianthis* (MidgeDam), and holes made by *Isophrictis* sp. (HoleDam). Damage scores were calculated as fractions (number of seeds in each category /total number of seeds scored per plant).

Leaf damage by herbivores was scored in mid-June and late-July on each plant. We scored percent cover of damage on three of the oldest leaves per plant caused by herbivores and calculated a damage index *D* for each. Herbivore damage classes included leaf-vascular-tissue feeders (SuckDam) and leaf chewers (ChewDam); *D* measures were calculated by summing *D* scores for component taxa. Stem and petiole damage by weevils (WeevilDam) and stem-boring larvae (StemBorer) were assessed in mid-June as the number of holes caused by each type of insect.

**REFERENCES**

Fick, S. E., and R. J. Hijmans. 2017. WorldClim 2: new 1-km spatial resolution climate surfaces for global land areas. International journal of climatology 37:4302–4315.

Hijmans, R. J., and J. van Etten. 2014. raster: Geographic data analysis and modeling. R package version 2:15.

Mitchell, N., G. L. Owens, S. M. Hovick, L. H. Rieseberg, and K. D. Whitney. 2019. Hybridization speeds adaptive evolution in an eight-year field experiment. Scientific reports 9:6746.

Whitney, K. D., R. A. Randell, and L. H. Rieseberg. 2006. Adaptive introgression of herbivore resistance traits in the weedy sunflower *Helianthus annuus*. The American Naturalist 167:794–807.

Whitney, K. D., R. A. Randell, and L. H. Rieseberg. 2010. Adaptive introgression of abiotic tolerance traits in the sunflower *Helianthus annuus*. New Phytologist 187:230–239.


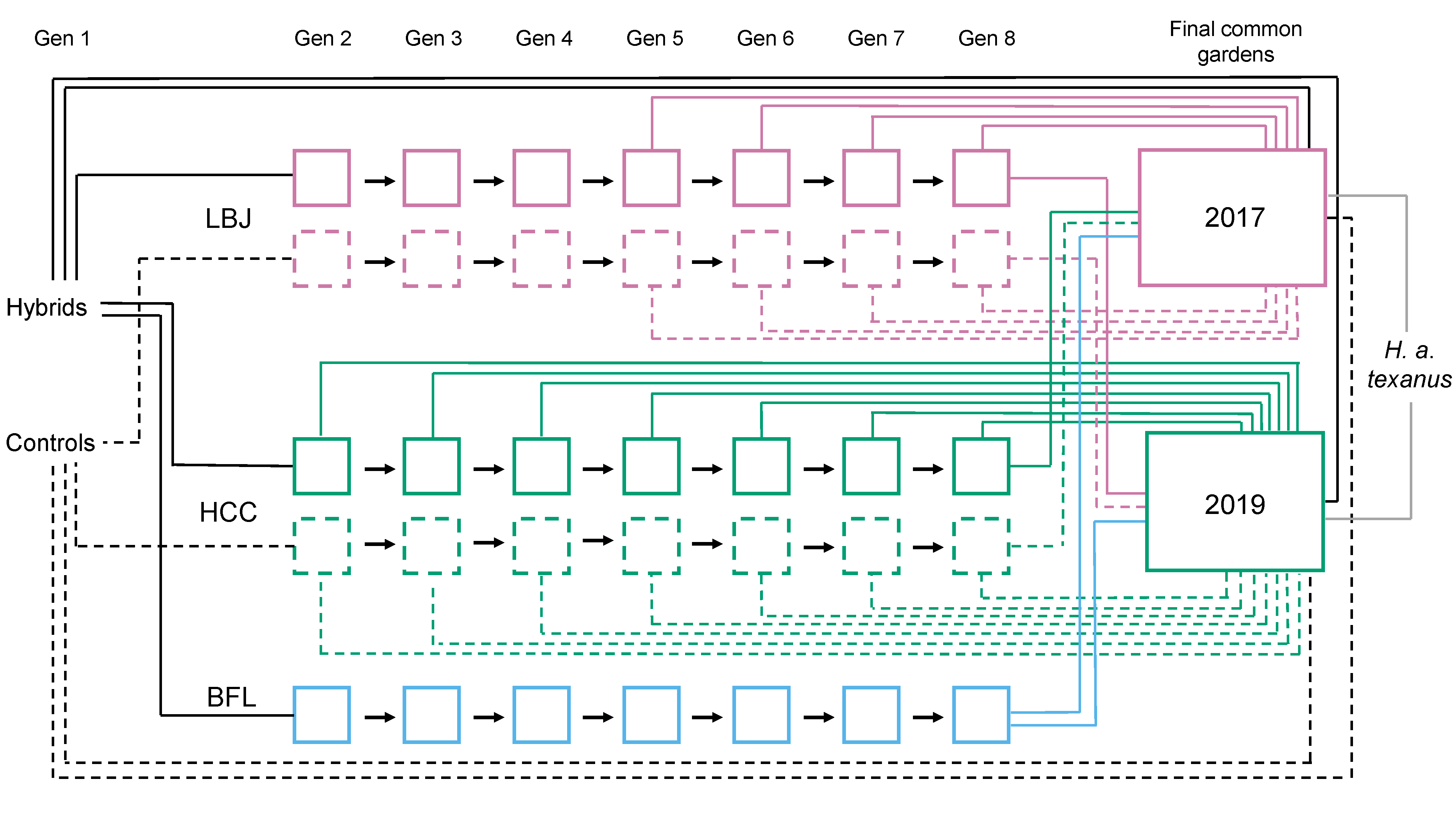


Figure S1. Experimental design for the current study. Boxes represent plots of plants grown in the field. Controls (dashed lines, *Helianthus annuus* ssp. *annuus*) and hybrids (solid lines) were synthesized as in the Methods and populations were established at three different home sites, LBJ (pink), HCC (green), and BFL (light blue). Only hybrids were established at BFL. Populations evolved in place through generation 8 and seeds were collected every generation. A final common garden was established in 2017 at LBJ with final generations of each treatment × home site combination, with intermediate generations for each (generations 2-4 had low germination rates and were not included), as well as individuals of the locally adapted taxon (gray, *H. a. texanus*). A similar setup was used in a final common garden in 2019 at HCC. See Fig. 1 for map of locations and Methods for details.


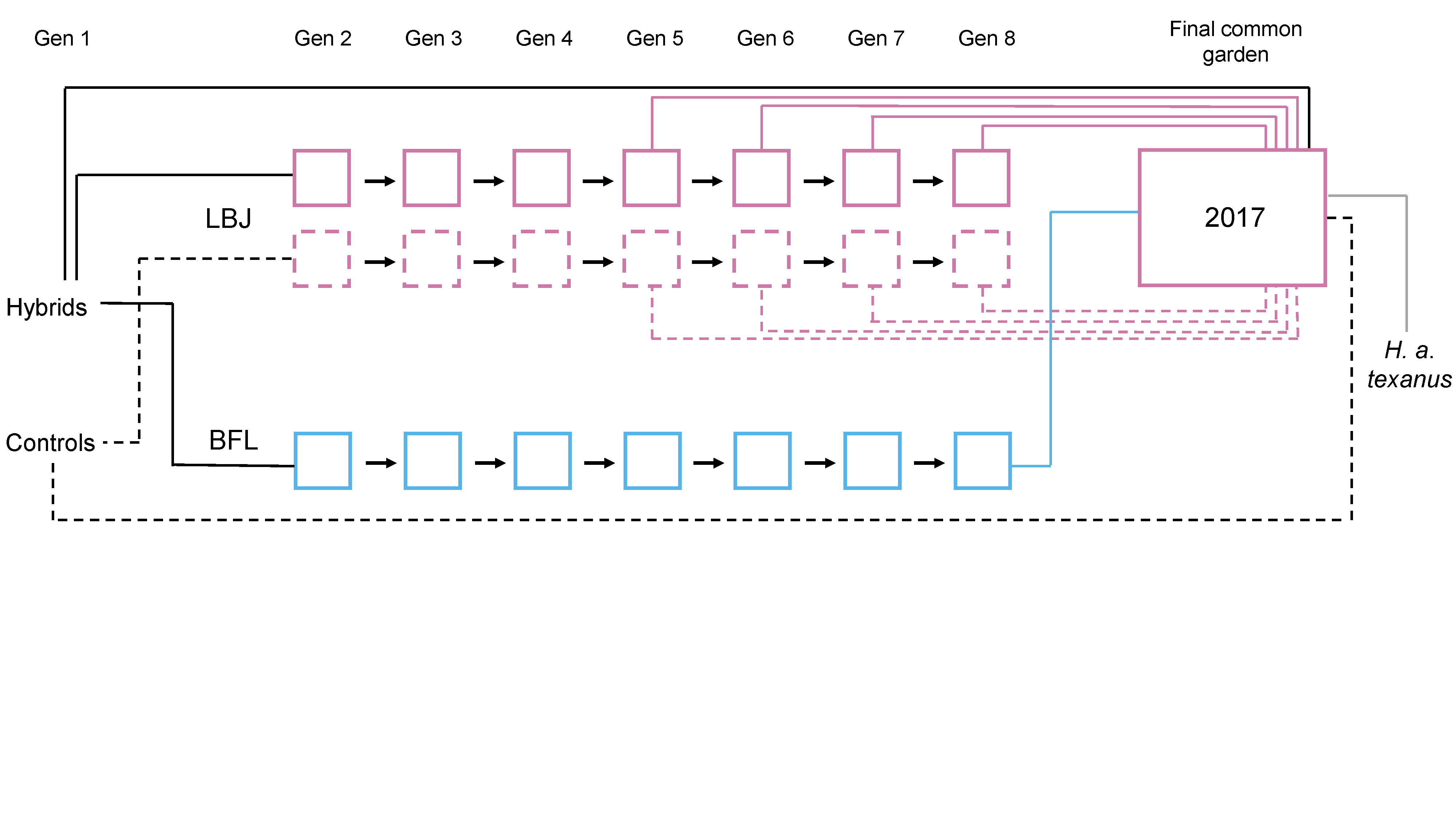


Figure S2. Experimental design for the 2017 common garden as reported in Mitchell et al. 2019. Graphic conventions as in Fig. S1.

Table S1. Climatic characterization of collection sites and common gardens.

| **Site Name** | **Species** | **Long** | **Lat** | **MAT (°C)** | **Mean Diurnal Range (°C)** | **Iso-therm-ality** | **Temp Season-ality (stdev × 100)** | **Max Temp Warmest Month (°C)** | **Min Temp Coldest Month (°C)** | **Temp Annual Range (°C)** | **Mean Temp Wettest Qtr (°C)** | **Mean Temp Driest Qtr (°C)** |
| --- | --- | --- | --- | --- | --- | --- | --- | --- | --- | --- | --- | --- |
| HCC | Common Garden | -95.04 | 29.39 | 20.6 | 9.0 | 34 | 605.9 | 32.6 | 6.5 | 26.1 | 27.6 | 16.6 |
| LBJ | Common Garden | -97.88 | 30.18 | 19.5 | 12.4 | 38 | 689.9 | 35.0 | 3.0 | 32.0 | 23.4 | 11.9 |
| BFL | Common Garden | -97.78 | 30.28 | 20.1 | 12.3 | 38 | 698.7 | 35.6 | 3.6 | 32.0 | 24.0 | 12.3 |
| LHR 1223 | *H. a. annuus* | -98.10 | 35.90 | 15.0 | 13.3 | 33 | 915.2 | 34.8 | -4.6 | 39.4 | 19.9 | 3.0 |
| RAR59 | *H. a. annuus* | -96.24 | 33.31 | 17.1 | 12.6 | 35 | 800.7 | 34.5 | -1.1 | 35.6 | 21.4 | 6.4 |
| K159 | *H. a. texanus* | -96.06 | 29.58 | 20.1 | 12.2 | 40 | 640.9 | 34.5 | 4.2 | 30.3 | 21.0 | 16.2 |
| K174 | *H. a. texanus* | -97.57 | 29.36 | 20.5 | 13.0 | 40 | 648.9 | 35.8 | 3.9 | 31.9 | 24.1 | 13.2 |
| K196 | *H. a. texanus* | -98.20 | 29.18 | 20.8 | 13.1 | 40 | 658.6 | 35.9 | 3.9 | 32.0 | 24.6 | 13.4 |
| RN | *H. debilis* | -94.90 | 29.25 | 20.8 | 7.8 | 31 | 604.7 | 32.2 | 7.4 | 24.8 | 27.8 | 20.5 |
|  |  |  |  |  |  |  |  |  |  |  |  |  |
| **Site Name** | **Species** | **Mean Temp Warmest Qtr (°C)** | **Mean Temp Coldest Qtr (°C)** | **MAP (mm)** | **Precip Wettest Month (mm)** | **Precip Driest Month (mm)** | **Precip Seasonality** | **Precip Wettest Qtr (mm)** | **Precip Driest Qtr (mm)** | **Precip Warmest Qtr (mm)** | **Precip Coldest Qtr (mm)** |  |
| HCC | Common Garden | 27.8 | 12.3 | 1158 | 160 | 60 | 26 | 374 | 213 | 337 | 256 |  |
| LBJ | Common Garden | 28.0 | 10.3 | 840 | 114 | 42 | 32 | 281 | 158 | 190 | 162 |  |
| BFL | Common garden | 28.7 | 10.8 | 809 | 110 | 39 | 32 | 270 | 154 | 179 | 161 |  |
| LHR 1223 | *H. a. annuus* | 26.7 | 3.0 | 756 | 122 | 21 | 46 | 292 | 81 | 235 | 81 |  |
| RAR59 | *H. a. annuus* | 27.1 | 6.4 | 1065 | 147 | 55 | 28 | 350 | 202 | 228 | 202 |  |
| K159 | *H. a. texanus* | 27.8 | 11.2 | 1059 | 126 | 59 | 23 | 315 | 198 | 271 | 223 |  |
| K174 | *H. a. texanus* | 28.3 | 11.6 | 850 | 110 | 44 | 33 | 288 | 154 | 205 | 159 |  |
| K196 | *H. a. texanus* | 28.7 | 11.8 | 703 | 94 | 31 | 32 | 225 | 125 | 174 | 132 |  |
| RN | *H. debilis* | 28.0 | 12.6 | 1064 | 163 | 54 | 30 | 364 | 195 | 304 | 246 |  |

Table S2. Fitness and 24 traits measured across both final common gardens. Abbreviations and units of measurement included.

|  | **Trait** | **Abbreviation** | **Unit** |
| --- | --- | --- | --- |
|  | Fitness | Fitness | Viable achenes/plant |
| Leaf/Ecophys | Specific leaf area | SLA | cm^2^ ∙ g^-1^ |
|  | Leaf dry matter content | LDMC |  |
|  | Leaf succulence | Succ | g ∙ cm^2^ |
|  | Leaf chlorophyll content | Chloro | SPAD reading |
|  | Leaf length:width ratio | LWR |  |
|  | Water-use efficiency | WUE | δ 13C |
| Pheno | Bud initiation time | DaysToBud | days |
|  | Seed maturation time | SMT | days |
|  | Plant longevity | Longevity | days |
| Architecture | Disk diameter | DiskDiam | mm |
|  | Plant volume | Volume | cm^3^ |
|  | Height of lowest branch | HtLow | cm |
|  | Relative branch diameter | RelBrDiam |  |
| Damage | Glandular trichome density | GlandDens | mm^-2^ |
|  | Nonglandular trichome density | HairDens | mm^-2^ |
|  | Leaf Carbon:Nitrogen | CNratio |  |
|  | Leaf-vascular-tissue damage | SuckDam | % |
|  | Leaf-chewing damage | ChewDam | % |
|  | Stemborer damage | StemBorer | Holes per plant |
|  | Weevil damage | WeevilDam | Petioles per plant *(Rhodobaenus* sp.) |
|  | Midge damage | MidgeDam | Fraction seeds killed (*Neolasioptera helianthis*) |
|  | Parasitoid damage | ParaDam | Fraction *N. helianthis* parasitized |
|  | Hole damage | HoleDam | Fraction seeds damaged by holes |
|  | Receptacle damage | RecepDam | Average number of receptacle holes *(Isophrictis* sp.) |

Table S3. Evolution of traits across gardens, homesites, and treatments. Values are the mean slope value from the Bayesian regression analyses. Homesite LBJ plants grown at the 2017 garden at LBJ, HCC plants grown at the 2019 garden at HCC, BFL plants (hybrids only) grown either at LBJ in 2017 or HCC in 2019.

|  |  | **LBJ 2017** | | **HCC 2019** | | **BFL 2017** | **BFL 2019** |
| --- | --- | --- | --- | --- | --- | --- | --- |
|  | Trait | Control | Hybrid | Control | Hybrid | Hybrid | Hybrid |
|  | Fitness | 0.001 | 0.154** | -0.025 | 0.041** | 0.115** | 0.087** |
| Leaf/EcoPhys | SLA | 0.023 | 0.146** | 0.116** | 0.122** | 0.036* | 0.088** |
|  | LeafLong | 0.000 | 0.013 | -0.009 | - | - | - |
|  | LDMC | 0.021 | -0.053* | -0.065* | 0.015 | -0.001 | 0.027 |
|  | Succ | -0.060** | -0.129** | -0.056* | -0.105** | -0.024 | -0.137** |
|  | Chloro | -0.064** | 0.060** | -0.028 | 0.075** | -0.022 | 0.100** |
|  | LWR | -0.002 | -0.132** | -0.041 | -0.004 | -0.048** | 0.071** |
|  | WUE | -0.025 | -0.079** | -0.084** | -0.087** | 0.051* | -0.144** |
| Pheno | DaysToBud | -0.003 | 0.149** | 0.033 | -0.035* | 0.074** | -0.039* |
|  | SMT | -0.092** | 0.035 | 0.073* | 0.012 | -0.114** | 0.110** |
|  | Longevity | 0.004 | 0.035 | 0.006 | 0.027 | 0.063** | -0.126** |
| Architecture | DiskDiam | -0.004 | 0.118** | 0.103** | 0.023 | -0.010 | 0.060** |
|  | Volume | -0.005 | 0.094** | 0.084** | 0.018 | 0.020 | -0.001 |
|  | HtLow | 0.013 | 0.145** | 0.020 | 0.041* | 0.039 | 0.017 |
|  | Bushy | 0.020 | 0.046 | 0.026 | - | - | - |
|  | RelBrDiam | 0.023 | -0.052* | -0.011 | -0.029 | -0.026 | -0.007 |
| Damage | GlandDens | 0.030 | 0.130** | 0.071* | 0.123** | -0.072** | 0.115** |
|  | HairDens | 0.070** | 0.016 | -0.001 | 0.051** | 0.023 | -0.015 |
|  | SuckDam | -0.069** | 0.033 | 0.042 | -0.010 | 0.008 | -0.035 |
|  | ChewDam | 0.002 | 0.070** | 0.082** | 0.038 | 0.004 | 0.039 |
|  | StemBorer | 0.007 | 0.028 | 0.081** | 0.019 | -0.013 | 0.009 |
|  | CNratio | 0.058* | -0.092** | -0.013 | -0.030 | -0.039* | -0.021 |
|  | WeevilDam | -0.009 | 0.023 | 0.081** | 0.047* | -0.003 | - |
|  | MidgeDam | -0.050** | -0.188** | -0.119** | -0.103** | 0.048** | -0.144** |
|  | ParaDam | -0.015 | -0.053* | 0.059* | 0.022 | -0.028 | 0.012 |
|  | HoleDam | -0.030 | -0.123** | -0.065* | -0.080** | 0.046** | -0.089** |
|  | GSW | 0.017 | -0.114** | -0.026 | - | - | - |
|  | RecepDam | -0.046* | -0.083** | -0.012 | -0.004 | 0.059** | -0.062** |

** = 95% credible intervals do not overlap zero

* = 80% credible intervals do not overlap zero

Table S4. Parallel versus non-parallel evolution - results from linear models on individual traits. Partial regression coefficients for Generation, Generation × HomeSite, the ratio of Generation/Generation × HomeSite, and Deviation from the 1:1 line. Results from the 2017 LBJ garden, 2019 HCC garden, or the "Combined" dataset, where each lineage is analyzed at its HomeSite common garden.

| **Type** | **Trait** | **Generation** | **Generation × HomeSite** | **Generation/**  **Generation × HomeSite** | **Deviation from 1:1** |
| --- | --- | --- | --- | --- | --- |
| **2017 (LBJ) Hybrid** | | | | | |
| Ecophysiology | SLA | 0.199 | 0.004 | 46.532 | 0.195 |
|  | LDMC | 0.028 | 0.004 | 6.299 | 0.023 |
|  | Succ | 0.139 | 0.014 | 9.862 | 0.124 |
|  | Chloro | 0.000 | 0.013 | 0.027 | -0.013 |
|  | LWR | 0.061 | 0.027 | 2.238 | 0.034 |
| Phenology | DaysToBud | 0.127 | 0.070 | 1.810 | 0.057 |
|  | SMT | 0.004 | 0.013 | 0.294 | -0.009 |
|  | Longevity | 0.014 | 0.002 | 6.431 | 0.011 |
| Architecture | DiskDiam | 0.047 | 0.019 | 2.502 | 0.028 |
|  | Volume | 0.016 | 0.007 | 2.373 | 0.009 |
|  | HtLow | 0.088 | 0.026 | 3.421 | 0.062 |
|  | RelBrDiam | 0.011 | 0.001 | 7.551 | 0.010 |
| Damage | GlandDens | 0.064 | 0.003 | 22.220 | 0.061 |
|  | HairDens | 0.021 | 0.020 | 1.043 | 0.001 |
|  | SuckDam | 0.008 | 0.001 | 6.980 | 0.007 |
|  | ChewDam | 0.064 | 0.004 | 17.894 | 0.060 |
|  | StemBorer | 0.005 | 0.004 | 1.214 | 0.001 |
|  | WeevilDam | 0.019 | 0.012 | 1.570 | 0.007 |
|  | MidgeDam | 0.217 | 0.011 | 20.109 | 0.206 |
|  | ParaDam | 0.000 | 0.035 | 0.011 | -0.035 |
|  | HoleDam | 0.111 | 0.007 | 16.460 | 0.104 |
|  | RecepDam | 0.022 | 0.018 | 1.220 | 0.004 |
| **2017 (LBJ) Control** | | | | | |
| Ecophysiology | SLA | 0.012 | 0.000 | 26.688 | 0.012 |
|  | LDMC | 0.000 | 0.000 | 41.007 | 0.000 |
|  | Succ | 0.031 | 0.001 | 27.476 | 0.030 |
|  | Chloro | 0.049 | 0.004 | 11.256 | 0.045 |
|  | LWR | 0.018 | 0.003 | 6.366 | 0.015 |
| Phenology | DaysToBud | 0.009 | 0.002 | 5.414 | 0.008 |
|  | SMT | 0.122 | 0.002 | 55.650 | 0.120 |
|  | Longevity | 0.009 | 0.007 | 1.290 | 0.002 |
| Architecture | DiskDiam | 0.000 | 0.000 | 1.308 | 0.000 |
|  | Volume | 0.001 | 0.005 | 0.141 | -0.004 |
|  | HtLow | 0.001 | 0.015 | 0.035 | -0.014 |
|  | RelBrDiam | 0.008 | 0.000 | 19.098 | 0.008 |
| Damage | GlandDens | 0.006 | 0.031 | 0.201 | -0.025 |
|  | HairDens | 0.020 | 0.020 | 0.999 | 0.000 |
|  | SuckDam | 0.021 | 0.000 | 101.462 | 0.021 |
|  | ChewDam | 0.001 | 0.001 | 0.806 | 0.000 |
|  | StemBorer | 0.003 | 0.000 | 6.699 | 0.002 |
|  | WeevilDam | 0.000 | 0.002 | 0.241 | -0.001 |
|  | MidgeDam | 0.002 | 0.019 | 0.087 | -0.017 |
|  | ParaDam | 0.005 | 0.015 | 0.362 | -0.010 |
|  | HoleDam | 0.005 | 0.000 | 29.007 | 0.005 |
|  | RecepDam | 0.003 | 0.006 | 0.470 | -0.003 |
| **2019 (HCC) Hybrid** | | | | | |
| Ecophysiology | SLA | 0.081 | 0.004 | 19.883 | 0.077 |
|  | LDMC | 0.020 | 0.000 | 206.248 | 0.020 |
|  | Succ | 0.278 | 0.007 | 37.285 | 0.270 |
|  | Chloro | 0.173 | 0.006 | 30.384 | 0.167 |
|  | LWR | 0.006 | 0.022 | 0.283 | -0.016 |
| Phenology | DaysToBud | 0.002 | 0.023 | 0.090 | -0.021 |
|  | SMT | 0.002 | 0.023 | 0.090 | -0.021 |
|  | Longevity | 0.097 | 0.042 | 2.327 | 0.055 |
| Architecture | DiskDiam | 0.028 | 0.003 | 10.312 | 0.025 |
|  | Volume | 0.004 | 0.024 | 0.185 | -0.019 |
|  | HtLow | 0.012 | 0.009 | 1.344 | 0.003 |
|  | RelBrDiam | 0.018 | 0.001 | 22.187 | 0.017 |
| Damage | GlandDens | 0.182 | 0.003 | 67.862 | 0.180 |
|  | HairDens | 0.007 | 0.002 | 3.790 | 0.005 |
|  | SuckDam | 0.008 | 0.005 | 1.411 | 0.002 |
|  | ChewDam | 0.027 | 0.005 | 5.908 | 0.022 |
|  | StemBorer | 0.000 | 0.001 | 0.013 | -0.001 |
|  | WeevilDam | 0.006 | 0.003 | 2.095 | 0.003 |
|  | MidgeDam | 0.219 | 0.013 | 17.141 | 0.207 |
|  | ParaDam | 0.004 | 0.001 | 3.318 | 0.003 |
|  | HoleDam | 0.000 | 0.018 | 0.003 | -0.018 |
|  | RecepDam | 0.017 | 0.009 | 1.982 | 0.008 |
| **2019 (HCC) Control** | | | | | |
| Ecophysiology | SLA | 0.015 | 0.001 | 27.760 | 0.014 |
|  | LDMC | 0.000 | 0.000 | 0.654 | 0.000 |
|  | Succ | 0.024 | 0.000 | 84.662 | 0.024 |
|  | Chloro | 0.015 | 0.003 | 4.195 | 0.011 |
|  | LWR | 0.002 | 0.015 | 0.126 | -0.013 |
| Phenology | DaysToBud | 0.018 | 0.020 | 0.879 | -0.002 |
|  | SMT | 0.057 | 0.006 | 9.176 | 0.051 |
|  | Longevity | 0.000 | 0.016 | 0.006 | -0.016 |
| Architecture | DiskDiam | 0.001 | 0.000 | 3.051 | 0.001 |
|  | Volume | 0.003 | 0.013 | 0.262 | -0.009 |
|  | HtLow | 0.000 | 0.004 | 0.062 | -0.004 |
|  | RelBrDiam | 0.005 | 0.004 | 1.156 | 0.001 |
| Damage | GlandDens | 0.000 | 0.023 | 0.002 | -0.023 |
|  | HairDens | 0.006 | 0.000 | 13.802 | 0.006 |
|  | SuckDam | 0.001 | 0.000 | 142.976 | 0.001 |
|  | ChewDam | 0.000 | 0.001 | 0.014 | -0.001 |
|  | StemBorer | 0.006 | 0.001 | 6.064 | 0.005 |
|  | WeevilDam | 0.004 | 0.004 | 0.983 | 0.000 |
|  | MidgeDam | 0.006 | 0.026 | 0.212 | -0.021 |
|  | ParaDam | 0.017 | 0.000 | 50.903 | 0.017 |
|  | HoleDam | 0.014 | 0.011 | 1.306 | 0.003 |
|  | RecepDam | 0.024 | 0.006 | 3.813 | 0.018 |
| **Combined** | | | | | |
| Ecophysiology | SLA | 0.190 | 0.022 | 8.620 | 0.168 |
|  | LDMC | 0.011 | 0.033 | 0.323 | -0.022 |
|  | Succ | 0.191 | 0.022 | 8.789 | 0.169 |
|  | Chloro | 0.039 | 0.033 | 1.188 | 0.006 |
|  | LWR | 0.027 | 0.072 | 0.373 | -0.045 |
| Phenology | DaysToBud | 0.109 | 0.083 | 1.317 | 0.026 |
|  | SMT | 0.006 | 0.012 | 0.522 | -0.006 |
|  | Longevity | 0.006 | 0.011 | 0.564 | -0.005 |
| Architecture | DiskDiam | 0.071 | 0.033 | 2.169 | 0.038 |
|  | Volume | 0.017 | 0.011 | 1.568 | 0.006 |
|  | HtLow | 0.067 | 0.021 | 3.209 | 0.046 |
|  | RelBrDiam | 0.019 | 0.007 | 2.785 | 0.012 |
| Damage | GlandDens | 0.107 | 0.002 | 44.398 | 0.105 |
|  | HairDens | 0.009 | 0.006 | 1.420 | 0.003 |
|  | SuckDam | 0.000 | 0.005 | 0.032 | -0.005 |
|  | ChewDam | 0.036 | 0.004 | 8.451 | 0.032 |
|  | StemBorer | 0.002 | 0.002 | 1.208 | 0.000 |
|  | WeevilDam | 0.020 | 0.026 | 0.772 | -0.006 |
|  | MidgeDam | 0.190 | 0.012 | 16.547 | 0.179 |
|  | ParaDam | 0.000 | 0.055 | 0.001 | -0.055 |
|  | HoleDam | 0.100 | 0.034 | 2.920 | 0.066 |
|  | RecepDam | 0.017 | 0.032 | 0.524 | -0.015 |
| Ecophysiology | SLA | 0.014 | 0.001 | 14.570 | 0.013 |
|  | LDMC | 0.000 | 0.000 | 13.000 | 0.000 |
|  | Succ | 0.032 | 0.002 | 13.786 | 0.030 |
|  | Chloro | 0.029 | 0.014 | 2.152 | 0.016 |
|  | LWR | 0.017 | 0.003 | 5.224 | 0.013 |
| Phenology | DaysToBud | 0.024 | 0.008 | 3.089 | 0.016 |
|  | SMT | 0.105 | 0.000 | 237.413 | 0.104 |
|  | Longevity | 0.002 | 0.001 | 2.300 | 0.001 |
| Architecture | DiskDiam | 0.000 | 0.000 | 0.652 | 0.000 |
|  | Volume | 0.001 | 0.006 | 0.087 | -0.006 |
|  | HtLow | 0.007 | 0.000 | 224.888 | 0.007 |
|  | RelBrDiam | 0.001 | 0.001 | 1.596 | 0.000 |
| Damage | GlandDens | 0.002 | 0.014 | 0.120 | -0.013 |
|  | HairDens | 0.020 | 0.007 | 2.894 | 0.013 |
|  | SuckDam | 0.001 | 0.006 | 0.219 | -0.005 |
|  | ChewDam | 0.001 | 0.000 | 72.350 | 0.001 |
|  | StemBorer | 0.000 | 0.008 | 0.043 | -0.008 |
|  | WeevilDam | 0.002 | 0.002 | 0.949 | 0.000 |
|  | MidgeDam | 0.000 | 0.042 | 0.011 | -0.042 |
|  | ParaDam | 0.007 | 0.000 | 14.221 | 0.006 |
|  | HoleDam | 0.000 | 0.005 | 0.000 | -0.005 |
|  | RecepDam | 0.000 | 0.024 | 0.001 | -0.024 |

Table S5. Principal component information for morphological analyses. Importance of components and component loadings for the 2017 LBJ and 2019 HCC common gardens.

|  | **LBJ (2017)** | | | | | | | | | | | |
| --- | --- | --- | --- | --- | --- | --- | --- | --- | --- | --- | --- | --- |
| **Importance of Components** | PC1 | PC2 | PC3 | PC4 | PC | PC6 | PC7 | PC8 | PC9 | PC10 | PC11 |  |
| Standard | 1.697 | 1.471 | 1.291 | 1.275 | 1.138 | 1.046 | 0.993 | 0.922 | 0.892 | 0.843 | 0.800 |  |
| Proportion | 0.161 | 0.121 | 0.093 | 0.091 | 0.072 | 0.061 | 0.055 | 0.047 | 0.044 | 0.040 | 0.036 |  |
| Cumulative | 0.161 | 0.282 | 0.375 | 0.465 | 0.538 | 0.599 | 0.654 | 0.701 | 0.746 | 0.785 | 0.821 |  |
|  |  |  |  |  |  |  |  |  |  |  |  |  |
| **Component Loadings** | PC1 | PC2 | PC3 | PC4 | PC | PC6 | PC7 | PC8 | PC9 | PC10 | PC11 |  |
| SLA | 0.261 | 0.403 |  |  |  | 0.153 |  | 0.363 | 0.217 |  |  |  |
| SMT |  | -0.179 |  |  | 0.178 |  | 0.271 | 0.306 | 0.217 | -0.315 | -0.19 |  |
| Longevity | 0.314 |  | -0.424 | -0.1 | 0.18 |  | -0.162 | 0.102 | -0.192 |  | -0.288 |  |
| DiskDiam | -0.139 | 0.14 | -0.145 |  | 0.143 | -0.19 |  |  |  |  |  |  |
| HtLow | 0.255 | 0.241 |  | 0.382 | 0.104 | -0.218 |  | -0.246 | 0.118 | -0.3 | 0.216 |  |
| RelBrDiam |  |  | 0.188 |  |  |  |  |  |  |  |  |  |
| SuckDam | -0.106 |  |  | 0.118 | 0.655 |  | 0.544 | -0.131 | -0.143 | 0.254 | -0.251 |  |
| StemBorer | -0.17 |  | -0.363 | -0.234 | -0.145 | 0.703 | 0.239 | -0.215 | 0.17 | -0.271 | 0.132 |  |
| Volume |  |  | -0.181 | -0.105 |  | -0.108 |  |  |  |  |  |  |
| GlandDens |  |  | -0.168 | 0.412 | -0.138 |  | 0.43 | 0.175 | -0.167 |  | 0.442 |  |
| HairDens |  |  | -0.287 | 0.161 | -0.564 | -0.25 | 0.325 |  | -0.207 |  | -0.455 |  |
| ChewDam |  | -0.142 | -0.322 |  |  | -0.195 |  |  | 0.692 | 0.533 |  |  |
| Weevil | -0.237 | -0.204 | -0.161 | -0.356 | 0.128 | -0.239 |  | 0.499 | -0.201 |  | 0.423 |  |
| Succ |  | 0.267 |  | -0.536 |  | -0.311 | 0.319 | -0.238 |  | -0.104 |  |  |
| DaysToBud | 0.288 |  | -0.455 |  | 0.15 |  | -0.115 | -0.172 | -0.136 |  | 0.168 |  |
| MidgeDam | 0.298 | -0.217 |  | -0.151 |  |  | 0.168 | -0.418 |  | 0.109 | 0.265 |  |
| HoleDam | 0.338 | -0.239 |  |  |  |  |  |  |  |  |  |  |
| RecepDam | 0.392 | -0.341 |  |  |  |  | 0.119 | 0.135 | 0.142 |  | -0.136 |  |
| ParaDam | 0.319 | -0.192 |  | -0.169 |  |  |  |  |  | -0.167 |  |  |
| LWR | 0.117 |  | 0.32 |  | -0.183 | 0.151 | 0.118 |  | -0.122 | 0.301 | 0.181 |  |
| LDMC | -0.207 | -0.489 | -0.102 | 0.258 |  |  | -0.2 | -0.155 | -0.186 |  |  |  |
| Chloro | -0.203 | -0.276 |  |  | 0.125 | -0.259 |  | -0.181 | 0.318 | -0.459 |  |  |
|  |  |  |  |  |  |  |  |  |  |  |  |  |
| **Importance of Components** | PC12 | PC13 | PC14 | PC15 | PC16 | PC17 | PC18 | PC19 | PC20 | PC21 | PC22 |  |
| Standard | 0.737 | 0.715 | 0.703 | 0.667 | 0.592 | 0.567 | 0.505 | 0.360 | 0.337 | 0.200 | 0.015 |  |
| Proportion | 0.030 | 0.029 | 0.028 | 0.025 | 0.020 | 0.018 | 0.014 | 0.007 | 0.006 | 0.002 | 0.000 |  |
| Cumulative | 0.851 | 0.880 | 0.908 | 0.932 | 0.952 | 0.970 | 0.984 | 0.991 | 0.998 | 1.000 | 1.000 |  |
|  |  |  |  |  |  |  |  |  |  |  |  |  |
| **Component Loadings** | PC12 | PC13 | PC14 | PC15 | PC16 | PC17 | PC18 | PC19 | PC20 | PC21 | PC22 |  |
| SLA | 0.14 | 0.109 | 0.373 | 0.221 |  |  |  |  |  |  | 0.573 |  |
| SMT | -0.236 | -0.511 |  | -0.305 | 0.168 | -0.163 | 0.284 | -0.112 |  |  |  |  |
| Longevity | 0.12 | -0.428 |  | 0.156 | -0.286 |  | -0.41 | 0.17 |  |  |  |  |
| DiskDiam |  | 0.187 | 0.287 | -0.201 | -0.367 | -0.664 | 0.165 | 0.302 |  |  |  |  |
| HtLow | -0.598 |  | -0.108 |  |  |  | -0.218 |  | 0.11 |  |  |  |
| RelBrDiam |  |  |  |  |  |  | -0.217 | 0.201 | -0.5 | -0.767 |  |  |
| SuckDam |  | 0.223 |  | 0.12 |  | 0.111 |  |  |  |  |  |  |
| StemBorer | -0.104 | 0.123 |  |  |  |  |  |  |  |  |  |  |
| Volume |  | 0.101 |  | -0.113 |  |  | 0.132 | -0.239 | 0.658 | -0.625 |  |  |
| GlandDens | 0.468 |  | -0.215 | -0.177 |  |  | -0.12 |  |  |  |  |  |
| HairDens | -0.144 |  | 0.277 | 0.172 |  |  |  |  |  |  |  |  |
| ChewDam |  |  |  | -0.122 | -0.133 |  |  |  |  |  |  |  |
| Weevil | -0.315 |  | 0.103 | 0.276 |  |  |  |  |  |  |  |  |
| Succ |  | -0.179 | -0.361 |  |  |  |  |  |  |  | 0.424 |  |
| DaysToBud |  |  |  | 0.166 | 0.182 |  | 0.514 | -0.236 | -0.419 | -0.111 |  |  |
| MidgeDam |  | -0.161 | 0.525 |  | 0.378 | -0.127 | -0.22 |  | 0.113 |  |  |  |
| HoleDam |  | 0.192 | -0.314 | 0.137 | 0.175 |  | 0.309 | 0.692 | 0.194 |  |  |  |
| RecepDam |  | 0.425 | -0.165 |  |  | -0.4 | -0.246 | -0.443 | -0.104 |  |  |  |
| ParaDam |  | 0.18 | 0.235 | -0.381 | -0.505 | 0.505 | 0.135 |  | -0.132 |  |  |  |
| LWR | -0.157 | -0.303 |  | 0.362 | -0.476 | -0.197 | 0.305 | -0.147 | 0.167 |  |  |  |
| LDMC | -0.13 |  |  | -0.141 |  |  |  |  |  |  | 0.701 |  |
| Chloro | 0.372 |  | 0.128 | 0.491 | -0.132 | 0.127 |  |  |  |  |  |  |
|  | **HCC (2019)** | | | | | | | | | | | |
| **Importance of Components** | PC1 | PC2 | PC3 | PC4 | PC | PC6 | PC7 | PC8 | PC9 | PC10 | PC11 |  |
| Standard deviation | 1.566 | 1.382 | 1.284 | 1.184 | 1.072 | 1.064 | 1.052 | 0.999 | 0.950 | 0.930 | 0.866 |  |
| Proportion of Variance | 0.133 | 0.104 | 0.089 | 0.076 | 0.062 | 0.061 | 0.060 | 0.054 | 0.049 | 0.047 | 0.041 |  |
| Cumulative Proportion | 0.133 | 0.237 | 0.327 | 0.403 | 0.465 | 0.527 | 0.587 | 0.641 | 0.690 | 0.737 | 0.778 |  |
|  |  |  |  |  |  |  |  |  |  |  |  |  |
| **Component Loadings** | PC1 | PC2 | PC3 | PC4 | PC | PC6 | PC7 | PC8 | PC9 | PC10 | PC11 |  |
| SLA | 0.100 | 0.247 | 0.189 | 0.626 | 0.323 |  |  |  |  |  |  |  |
| SMT | 0.166 |  | -0.236 | -0.123 | 0.120 |  | 0.325 | 0.144 | 0.113 | 0.363 | -0.456 |  |
| Longevity | -0.324 | 0.372 | -0.240 |  | -0.206 |  | 0.123 |  | 0.155 | 0.116 | 0.257 |  |
| DiskDiam | 0.261 | 0.190 | -0.116 | -0.183 | 0.193 |  |  |  |  |  |  |  |
| HtLow | -0.226 |  | -0.278 |  | 0.380 | 0.410 |  | -0.244 | 0.365 | -0.228 | -0.183 |  |
| RelBrDiam |  | -0.166 |  |  |  |  |  |  |  |  |  |  |
| SuckDam |  |  | 0.241 | -0.161 | 0.325 | 0.231 | -0.548 |  |  |  |  |  |
| StemBorer |  |  |  |  | -0.158 |  |  | 0.217 |  |  |  |  |
| Volume | 0.120 | 0.317 | -0.113 |  |  | -0.154 |  |  |  | 0.129 | 0.133 |  |
| GlandDens |  |  | -0.284 | 0.183 |  | 0.311 | -0.176 | 0.143 | -0.455 | 0.112 | -0.290 |  |
| HairDens |  | 0.120 | -0.156 |  |  | 0.230 | -0.289 |  | -0.490 | 0.119 |  |  |
| ChewDam |  | 0.182 | -0.286 | 0.147 |  | -0.321 |  | 0.157 | -0.154 | -0.714 | -0.162 |  |
| Weevil |  | 0.115 | 0.159 |  | -0.176 |  | -0.199 | 0.747 | 0.347 |  | -0.163 |  |
| Succ |  | 0.329 | 0.304 | -0.474 | -0.212 |  | 0.252 | -0.131 | -0.166 |  | -0.296 |  |
| DaysToBud | -0.426 | 0.246 | -0.171 |  |  | 0.221 |  |  | 0.141 |  |  |  |
| MidgeDam | -0.293 |  | 0.270 | -0.306 | 0.191 |  |  | 0.114 | -0.197 | -0.266 | 0.234 |  |
| HoleDam | -0.263 |  |  |  | 0.163 | -0.486 | -0.124 |  | -0.164 | 0.277 | -0.113 |  |
| RecepDam | -0.366 |  |  |  |  | -0.370 | -0.366 | -0.223 | 0.194 |  | -0.447 |  |
| ParaDam | -0.169 |  |  | -0.177 | 0.578 | -0.163 | 0.260 | 0.388 | -0.243 | 0.135 | 0.114 |  |
| LWR | -0.228 | -0.389 | 0.129 | 0.123 |  |  | 0.204 |  |  | -0.173 | -0.321 |  |
| LDMC | -0.114 | -0.471 | -0.401 | -0.206 | -0.133 |  | -0.191 |  |  |  | 0.205 |  |
| Chloro | 0.355 |  | -0.293 | -0.214 | 0.127 | -0.142 | -0.221 |  |  | -0.157 |  |  |
|  |  |  |  |  |  |  |  |  |  |  |  |  |
| **Importance of Components** | PC12 | PC13 | PC14 | PC15 | PC16 | PC17 | PC18 | PC19 | PC20 | PC21 | PC22 |  |
| Standard deviation | 0.842 | 0.829 | 0.767 | 0.703 | 0.688 | 0.634 | 0.559 | 0.486 | 0.398 | 0.182 | 0.014 |  |
| Proportion of Variance | 0.039 | 0.037 | 0.032 | 0.027 | 0.026 | 0.022 | 0.017 | 0.013 | 0.009 | 0.002 | 0.000 |  |
| Cumulative Proportion | 0.816 | 0.853 | 0.885 | 0.912 | 0.938 | 0.960 | 0.977 | 0.990 | 0.998 | 1.000 | 1.000 |  |
|  |  |  |  |  |  |  |  |  |  |  |  |  |
| **Component Loadings** | PC12 | PC13 | PC14 | PC15 | PC16 | PC17 | PC18 | PC19 | PC20 | PC21 | PC22 |  |
| SLA | 0.122 |  | 0.130 |  |  |  |  |  |  |  | 0.584 |  |
| SMT | -0.272 | 0.172 | 0.331 |  | 0.401 | 0.131 |  |  |  |  |  |  |
| Longevity |  | 0.218 |  | 0.137 |  | -0.206 | 0.255 |  | -0.538 | 0.210 |  |  |
| DiskDiam | 0.160 |  |  | -0.745 | -0.120 | -0.396 |  | 0.127 |  |  |  |  |
| HtLow |  | -0.332 |  |  |  | 0.201 | 0.275 | -0.167 |  |  |  |  |
| RelBrDiam |  |  |  |  |  |  |  | 0.319 | -0.539 | -0.735 |  |  |
| SuckDam | -0.542 | 0.210 |  | 0.157 |  | -0.221 |  |  | -0.137 |  |  |  |
| StemBorer |  | 0.317 | 0.322 | -0.264 | -0.535 | 0.517 | 0.239 | -0.110 |  |  |  |  |
| Volume |  | 0.132 |  |  |  |  | 0.185 | -0.468 | 0.337 | -0.624 |  |  |
| GlandDens |  |  |  |  |  |  | -0.374 | -0.456 | -0.169 |  |  |  |
| HairDens |  |  |  |  | 0.214 |  | 0.546 | 0.390 | 0.211 |  |  |  |
| ChewDam | -0.350 |  |  |  | 0.149 |  |  |  |  |  |  |  |
| Weevil | 0.166 | -0.344 | -0.108 |  |  |  | 0.102 |  |  |  |  |  |
| Succ |  |  | -0.113 | 0.145 | -0.171 |  |  |  |  |  | 0.488 |  |
| DaysToBud |  | 0.111 | 0.192 |  | -0.167 | -0.155 | -0.446 | 0.392 | 0.402 | -0.143 |  |  |
| MidgeDam | 0.246 |  | 0.395 | -0.180 | 0.456 | 0.143 |  | -0.209 |  |  |  |  |
| HoleDam | -0.159 | -0.543 | 0.385 |  | -0.211 | -0.107 |  |  |  |  |  |  |
| RecepDam | 0.190 | 0.319 | -0.273 | -0.107 | 0.184 | 0.149 |  |  |  |  |  |  |
| ParaDam |  | 0.162 | -0.422 |  | -0.207 |  |  |  |  |  |  |  |
| LWR | 0.196 | 0.235 | 0.144 | 0.126 | -0.106 | -0.567 | 0.275 | -0.138 | 0.150 |  |  |  |
| LDMC |  |  |  | -0.122 |  |  |  |  |  |  | 0.648 |  |
| Chloro | 0.485 | 0.126 | 0.314 | 0.448 | -0.183 |  | -0.130 |  | -0.107 |  |  |  |

Table S6. Tests of phenotypic parallel divergence. Values for pairwise angles in degrees (*θ*_P_) of phenotypic divergence vectors between generation 1 and generation 8 hybrids at the 2017 LBJ garden (upper table) and 2019 HCC garden (lower table), for all pairwise comparisons at that garden Within each comparison, lower left triangles contain observed values, upper right triangles contain associated p-values generated from 1000 bootstrap replicates.

| 2017 LBJ | | | | | |
| --- | --- | --- | --- | --- | --- |
|  | HCC hybrid | LBJ hybrid | BFL hybrid | HCC control | LBJ control |
| HCC hybrid | . | **< 0.001** | **< 0.001** | 1.000 | **0.034** |
| LBJ hybrid | **37.1°** | . | **< 0.001** | 1.000 | 0.190 |
| BFL hybrid | **43.0°** | **40.9°** | . | 1.000 | 0.464 |
| HCC control | 79.4° | 87.0° | 88.6° | . | **0.006** |
| LBJ control | **47.6°** | 68.6° | 74.9° | **66.3°** | . |
|  |  |  |  |  |  |
| 2019 HCC | | | | | |
|  | HCC hybrid | LBJ hybrid | BFL hybrid | HCC control | LBJ control |
| HCC hybrid | . | **< 0.001** | **< 0.001** | 1.000 | 0.454 |
| LBJ hybrid | **39.4°** | . | **< 0.001** | 1.000 | 1.000 |
| BFL hybrid | **38.1°** | **22.8°** | . | 1.000 | 0.802 |
| HCC control | 93.3° | 117.2° | 122.6° | . | *0.074* |
| LBJ control | 70.8° | 79.5° | 75.9° | 78.0° | . |
